# Supplementary material for: Revisiting the role of Dcc in visual system development with a novel eye clearing method
Source: eLife. 2020 Feb 25;9:e51275. doi: 10.7554/eLife.51275 (PMC7062470; doi:10.7554/eLife.51275)
Supplement: Supplementary file 1. [file elife-51275-supp1.docx]

| **Primary antibodies** | | | | | |
| --- | --- | --- | --- | --- | --- |
| **Antigen** | **Species** | **Catalog #** | **Company** | **Dilution** | **Immunohistochemistry** |
| Islet1 | Rabbit | Ab20670 | Abcam | 1:300 | cryosections/whole-mount |
| Chx10 | Sheep | X1180-P | Exalpha | 1:300 | cryosections |
| Calbindin | Mouse | 300 | Swant | 1:1000 | cryosections |
| Chat | Goat | Ab144p | Abcam | 1:500 | cryosections/flat-mount |
| Rbpms | Rabbit | 1830-RBPMS | Phosphosolution | 1:500 | cryosections/flat-mount/whole-mount |
| Dcc | Goat | Sc-6535 | Santa Cruz | 1:500 | cryosections/whole-mount |
| Sox2 | Rabbit | Ab97959 | Abcam | 1:500 | cryosections |
| Crx | Mouse | H00001406-M02 | Abnova | 1:2000 | cryosections |
| ßIII-tubulin | Mouse | MMS435P | Covance | 1:500 | cryosections |
| Pax2 | Rabbit | 7160000 | Life Technologies | 1:300 | whole-mount |
| Pax2 | Goat | AF3364 | R&D systems | 1:300 | whole-mount |
| Tag1 | Goat | AF4439 | R&D systems | 1:500 | cryosections/Whole-mount |
| Collagen-IV | Goat | 134001 | Bio-Rad | 1:400 | whole-mount |
| Opn1-sw | Goat | Sc-14363 | Santa Cruz | 1:1000 | whole-mount |
| OPN1MW | Rabbit | AB5405 | Merck | 1:300 | flatmount |
| Laminin | Mouse | Ab11575 | Abcam | 1:300 | cryosections |
| dsRed | Rabbit | 632496 | Clontech | 1:300 | whole-mount |
| Rhodopsin | Mouse | MABN15 | Millipore | 1:500 | cryosections |
| Recoverin | Rabbit | AB5585 | Millipore | 1:1000 | cryosections |
| Opn1-mw | Rabbit | AB5405 | Millipore | 1:300 | cryosections/Whole-mount |
| Arrestin-C | Rabbit | Ab15282 | Abcam | 1:1000 | cryosections/Whole-mount |
| **Secondary antibodies** | | | | | |
| Anti Rabbit Alexa 488 mcr | Donkey | 711-545-152 | Jackson Immunoresearch | 1:500 | cryosections/Flat-mount/whole-mount |
| Anti Rabbit cy3 mcr | Donkey | 711-165-152 | Jackson Immunoresearch | 1:500 | cryosections/Flat-mount/whole-mount |
| Anti Rabbit Alexa 647 mcr | Donkey | 711-605-152 | Jackson Immunoresearch | 1:500 | cryosections/Flat-mount/whole-mount |
| Anti Goat Alexa 647 mcr | Bovine | 805-605-180 | Jackson Immunoresearch | 1:500 | cryosections/Flat-mount/whole-mount |
| Anti Goat Alexa 488 | Donkey | A11055 | Life Technologies | 1:500 | cryosections/Flat-mount/whole-mount |
| Anti Goat Alexa 555 | Donkey | A21432 | Life Technologies | 1:500 | cryosections/Flat-mount/whole-mount |
| Anti Goat cy3 mcr | Donkey | 705-165-147 | Jackson Immunoresearch | 1:500 | cryosections/Flat-mount/whole-mount |
| Anti Sheep cy3 mcr | Donkey | 713-165-1471:500 | Jackson Immunoresearch | 1:500 | cryosections/Flat-mount/whole-mount |
| Anti Sheep Alexa 647 mcr | Donkey | 713-605-147 | Jackson Immunoresearch | 1:500 | cryosections/Flat-mount/whole-mount |
| Anti Sheep Alexa 488 | Donkey | A11015 | Life Technologies | 1:500 | cryosections/Flat-mount/whole-mount |
| Anti mouse Alexa 488 | Donkey | A21202 | Life Technologies | 1:500 | cryosections/Flat-mount/whole-mount |
| Anti mouse Alexa 647 mcr | Donkey | 715-605-150 | Jackson Immunoresearch | 1:500 | cryosections/Flat-mount/whole-mount |
