# Supplementary material for: Revisiting the role of Dcc in visual system development with a novel eye clearing method
Source: eLife. 2020 Feb 25;9:e51275. doi: 10.7554/eLife.51275 (PMC7062470; doi:10.7554/eLife.51275)
Supplement: Supplementary file 3. [file elife-51275-supp3.docx]

**Mus musculus phosphodiesterase 6B, cGMP, rod receptor, beta polypeptide (Pde6b), mRNA**

NCBI Reference Sequence: NM_008806.2

#### Genomic chr5 :

tccgggtctc tgagcagaga caggttgggc cactcatgtg gtatgagtca 108388322

gctgcctccc ctgggctccc taatcatcac taagaaagac tttgctgaca 108388372

ACAGGGTTTG CAGAATCCAT GTGTACCTGG GGGTGGAGAA AGCCCCGGGC 108388422 ex1

ACAGCAGCAG GAACACC**ATG** AGCCTCAGTG AGGAACAGGT ACGCAGCTTC 108388472

CTGGATGGGA ACCCCACGTT TGCCCATCAA TACTTTGGGA AGAAGTTGAG 108388522

CCCTGAAAAT GTGGCAGGGG CCTGTGAAGA TGGTTGGCTG GCGGACTGTG 108388572

GCAGCCTGCG AGAGCTGTGC CAGGTGGAAG AGAGTGCAGC ACTTTTTGAA 108388622

CTGGTGCAGG ACATGCAGGA GAGTGTCAAT ATGGAACGTG TGGTCTTCAA 108388672

GATCCTGCGG CGCCTCTGCA CCATCCTGCA TGCCGACCGC TGCAGCCTCT 108388722

TTATGTACCG CCAGCGCAAT GGCATAGCTG AACTTGCTAC GCGGCTCTTC 108388772

AGCGTGCAGC CTGACAGCCT TCTGGAGGAT TGCCTGGTGC CCCCTGACTC 108388822

TGAGATCGTC TTCCCCCTGG ACATTGGGAT TGTGGGCCAT GTGGCTCAGA 108388872

CCAAGAAGAT GATAAACGTG CAGGATGTGG CAGAGgtggg tgttctccag 108388922

ctatggtgca gaggtgtgtt ctgcctcagc atccagagat ttcacaagga 108388972

cttcaggtcc agagccaagg tccttgcagc tcacccttgc ctgtcctatc 108389022

tcctcaccgt gcttagcctc ctgtcttttc cttccaggac tagtttttcc 108389072

agtttaaggt gggggggggg gggcttcaag agcgctttaa agtgtccctc 108389122

ctgttctccc ccaaactgcc taattgccac agaaacacta tttaagagac 108389172

tttcttgtgg aggcttttca gagtcagcct ctggccttgc caaggtcagg 108389222

ccttctttat gcatgataag cactgtgagg acccagggca gcttcagtga 108389272

tcatcaggtg tgaagagtct tccatggata actatggttg tctgcgtaga 108389322

ggtctgaagt gatcaggcca gggctggtat tgaggccaga tgccccaaat 108389372

ccccagccat ggcagaagca gctttaccca caaagggaac cacgctggtc 108389422

ctggagacag aggatctggg atctaggaga ggatgggaat ggagttacaa 108389472

gagattcagg gtccaggttg ggaatggatt tattgacaca gacttcatgg 108389522

aagctgctgg tttgtggcag gtataaccag gacagagctc ctatgggtgg 108389572

gaagcactgt gggacacagt gagtgaggta cccgtagata tgggtgtgac 108389622

aacagccagg gaggctcaga aaggggtcaa aaatgtattc catggattag 108389672

ggagctctac agagggttgg aggacagtct tcctctcctt gcctgttctg 108389722

tttcttttcc tctttcttcc ccaagatacc caggttgcct cagcacctgc 108389772

tacataggtc aacaacatgt agtaggggaa tcccagaatg ggagaaggtc 108389822

ccctcttgga cagattcccc agaggcagtg tgctaccttc actcctggcc 108389872

aagtgggaag gttgggcagg agggtgctgc aatcctctta tacttatctg 108389922

ttataaacag gcccctgccc agcgacctga atagaataat ctgcaggatt 108389972

atcttggcct tcactttgct tcctttttaa gaaatgggtc atccatgagc 108390022

caaatggagc ataaattacc aacaaagcaa gaaatttggt atatagggga 108390072

ggctgtgaca gatcattaat tctggggcgc atggtctacc cacccttcct 108390122

aatttttctc acgcttcctc ctgccttctt tcattcattc ccaccttacc 108390172

cttttcttcc ccctcccacc caccttcagg gaatcttcaa agcagtgcct 108390222

acacgtagcc ctgagcctga ggcaggttct ctcctttcta ttgccctgat 108390272

ccacaccaaa gttgctctgt ggtgttgctc tgcggtaaga tgaagttgca 108390322 gagttgtcct taattctatt cccaggccta cccatgtcct acagcccctc 108390372

tccaaggttt atagtcactc tgtggcctca aagatacatc cttggtggca 108390422

catcatgtct actactgtta tgatgattct gtgacctgca ggttgtgcag 108390472

gtttgtattg atcccagact gagggaaaag gagtaaccgt caaagtcagc 108390522

aaaatccata tttccaccag tggcatcagg acactctgca tcgccaagga 108390572

cagaatactg ggttcacatg caggtttgca gcagagaggc aaactgaggc 108390622

agtcagcaca gggtctcccc aacacactag gtgctttcct tgcaaatggt 108390672

gagccacatg gtgggcctct gtggattgca gtgttgttct atagtgtggt 108390722

agatacaaca acacaggact aacacaaagt tttagttgca catagatact 108390772

cacagcacct ggatacactg atctccaatt tttgctgaga aaatgatgtc 108390822

tgcttcacca tgtagctgca gtagtcacac cagactgcta ctaagcataa 108390872

agaaatccta aacaaaaaca gatactgaac acatttaaac catgaacata 108390922

ggtacaggta taccaggagt acaggaagac cttgaggacc caaggatcat 108390972

gagcacaggc agaacatgaa cacaagtagg acatgcccac aggtaggaca 108391022

tgatcacaga acagaataca gatacactct gcctaaagca aatgttagta 108391072

agtatgggaa aatggctccc ctgagccctg agtgtctatg ttcttggcat 108391122

tgaggactgc taacatttct gatgcgatgt atgtgtccga aacctcatgc 108391172

ccccttctaa gcagaagcat gccccaggat gtggctttgt ggagggaaag 108391222

tctctttgag gctatgctca tagatccagc ctgtagggaa tgtggtagta 108391272

ttggaatgtg tgggacatac ctctgagcag agacctgagt ccatggcctg 108391322

agactagctc tctcaggacc ctataacttc atatgtgagt gtcacagctt 108391372

tgccggagga acagaggcta aaacctgaat aatagatatt tcactagacc 108391422

tcagggtcat tgtaaccagt tcagaacaga ataagccctc gtatttcaaa 108391472

accatacacc caaaagccca gaaaacccgg gagatagaag ctgggctcta 108391522

tgtcttgcac tcaatatgtc ataattcaac aaaacggcat gatcatctgc 108391572

cttgcctgtc ttctggaaag gtctgttagc attaaggaga ccagaactgc 108391622

acacatatca atccaaagta caagtggaga gaaattcacc aaagaataat 108391672

gatatttatt tatttatctc aatagcagtg agaatctctc tgttctggca 108391722

aactattaac atatttaagg aagttggggc aaggggtaga tttcaaagcc 108391772

aaaagaaaaa aaaaaagtaa gttacttcca taaaaagttc attggtctta 108391822

gggatcaaag gcaggaactg tcagcagctc actggtagag gcacccatgc 108391872

aggacaggta ttctcttcag ggtgtgttaa atgaattgtt ttgattttag 108391922

ggaccattag agataaactt tgggtaggtg tggtggtgca tgtctttact 108391972

cccagaactg caggtgtggt ggtgcgtgcc tttaaaccca ggactttgtc 108392022

tggttccagg ctaaccaagg ttatatggta atagcctggc tcaaaaaaca 108392072

aacaaacaaa caaacaaaca aacaaacaaa aaccatagat tttgctactg 108392122

aaattttttc atatgttaga aagtatggcg gaagaagtat ctctggggtc 108392172

tttggaaagt ccttaagaaa gtatttatca gctggatggt ggtggcacac 108392222

atctttaatc ccagcatgca agaagcaaag acaggtgatt ctctatgagt 108392272

ttgaggccag cctggtctac agagcaagtt tcagaacagt cagggcagtt 108392322

ataaagagaa caaaatattt ttaaaaacta tttgtctcag aggagcaaac 108392372

atgagtgtgc tccccacgcc cacatccttt cattacatcc tagatccaat 108392422

tatttcttgg atttaacaag aattatgtca tcctttaatc attaattttt 108392472

atatttttcc ctctgatcaa gaacattctt caaatcattg ctgaccaaat 108392522

aacattgtat cctttgatgc tgagaggggc ctgtactgat tggctgcatg 108392572

tgtgggtgag tcggttggct gttgagagaa gagtttaata gctagacatc 108392622

agtgccaagg ccatttcagt cctatttaac caacagaaag cagaaacaca 108392672

gagggtgaag ctctcgggac cggtgccctg ccctgtcacg aagctccatc 108392722

cccccaggcg ctggctgtta ccacactggg tcaactgtca ttttgttaga 108392772

tgctaaattt ctatccactc ttgacagaca gcagctacaa ttttaagata 108392822

agcaatacaa agtttggggc atttttcttt tatagtgttt taaattgatt 108392872

tatttatatg agtatgctat cactgtcttc agacacatca gaagagggca 108392922

tcggaaccca tttcagatgg ttgtgagcca ccatgtggtt gctgggaatt 108392972

gaactcagga cctctggaag agcagttggt gctaactgct gagccatctc 108393022

tccagccccg tacaaagtat tgttaacaat aatatgacaa cttccgtttc 108393072

atgttttttt ttaatggaga aagtctatta ttttactttt aataaaatac 108393122

ggtaaaacaa atatgaatag gaaaaaacaa cgaaagaaga aaaagagcca 108393172

aaaacataag gaatatatgt agatgcagag acacatgtgt ttgcacacac 108393222

aggaattcca taaaaacaca aaactgtaag tcctaatata tatgtaaagg 108393272

acctgcaagg ttaaaaaaat atatatcctg gcttaacctt atgaaacaaa 108393322

gaacctccac acttgatctt agccaaaagg ctgagaagca ataacaaaga 108393372

acctccaaag aagctgttga gttcattttc tgttggccat ctactgctgg 108393422

gcgtggggcc taccctcaag gatagtctgt ttcctcagtg agacttcctt 108393472

ggagaaaact aaaaataacc ttcatttgca agtggctatc agttggagat 108393522

agcttctggg ttaggaatga aggcatgtgt cctcttctcc ttttagttca 108393572

aggaccccac tcaggaagag atccacacag gcactgtgtg tgctgacaca 108393622

gtctttgtga gttcatatgt gtgccagcct tgttatgtct agaaggcctt 108393672

aattccttgg tgctcaccat cctttctggg tcttacactc tttctacccc 108393722

atcttccaca gagtttcctg agctctgagg ggagggattt gatgaaacat 108393772

ccctttagag ctgagtgttc caatctccct ctcccctccc cctccccctc 108393822

cccctctccc tctccctctt cccctcccta tccctctccc cctccctctt 108393872

cctcgccccc tccttctccc cctctccacc tccccctctc cctctctccc 108393922

tccttctccc cctctctccc ccctctccct ctcccccttc tcctccccct 108393972

gcacactgtc tatgggtctc tgtatttatt cccatctgct acaggatgag 108394022

gcttctctca taactgctga gcaggtctat gagtgaagca aaatgttgtt 108394072

tggcatctat tacacaaacc taagctcaaa gggtaaccaa tgaaacaaat 108394122

cagatgacca cagaggagtt tgtgggactt actatggtaa ctgagccttt 108394172

cccttgtttt gtggggctgg ttttgttttg ttttgttttg ttttgttttg 108394222

ttttgttttg ttttgttttg ttttgttttg ttttgttttg tttttcaaga 108394272

caagattttt ctgtgtagcc ctggctgtcc tggaactcac tttgtagacc 108394322

aggctagcct tgaactcaga aatccacctg cccctgcctc ctgactgctg 108394372

gtattaaagg tgtgtgccac cacgcctggc ttgtatgtat atcttaacaa 108394422

tatcatcatc cttctcacaa agaaaagtaa gggtcagagt cttatgatat 108394472

ggtcttattc tgactgtagt tttttcatac tcttttgcca tttctgcttt 108394522

acctgtgaca tcagaccatt ttcaagactc tatatttgaa atttgcatca 108394572

agcggtactg ctctagctct gtagatcttt agaaaagagt aattgttgac 108394622

ctcaagaatt gaaagcaaga aaattaaaag gtagtgagca gttatagtat 108394672

aataacagat attttttctt tagaagtaat tcttttagct acactgattt 108394722

ttttttccaa aaagatttgt taggtaagtt tcattttatc aaatttgact 108394772

tatacaaata caattagaga aatgatttct catataaata atctcagaca 108394822

tacgaacctt gaagcaagaa agccaagagg cagtaatccc caatcccctg 108394872

gtccccagac ctactattat aacttgataa ttggaaaagt agggcactca 108394922

aagccagaat tctgtgcaca gtttaaaatg tgtttttcag tttcaagaca 108394972

acttctatga gaacacattt tgctgactct atgcaaagca atcacaggat 108395022

cagaaaaaaa tagaaaaacc acaggtcctc cttcaacttt ggaggaccca 108395072

aataagggaa aaggaaacac tgccagcatt gcttataaaa atgggttctg 108395122

ttacatttct acaagctgta tagagcttaa gaaagttttc ttagatctag 108395172

gaaagaatct aagagccaac aatgtttcaa ataagacaaa aaaaaatatc 108395222

caccggaagt gttggcgctt gcctttagtc ccagcactta ggaggcagag 108395272

agagttagat ctctgagctt gaggccagct tggtctacga agtgagttcc 108395322

aggacagaca gccctatgaa gatcgagaga gaccctgtct caaaacaaca 108395372

aaccttgtta aaagctgttt tagatgtttt ctatgtaata gaaacacaca 108395422

cacacacaca cacacacaca cacacacaga gagagagaga aattgtcttc 108395472

acaaaatata agacccatgt ttcctagaga agttatgaaa agaaagaagg 108395522

atgctacatt tgttgcattt taaagggaag caattgagaa cagcttggaa 108395572

gatccccggt ggaccagtgt ggaagccatc agatgctgga ggagtatata 108395622

caaactacat ctgtgcaaca tgtgtgtaca tcatgctacc aaggatgcaa 108395672

accatgatta gttgcctcga acaggggcat ggtggaactg tcttgttata 108395722

cagggctcaa acatattagg gaagccccaa agaaaacctt tcattctgga 108395772

cctttttttt tttttttttt ttttttttgg gttttccaga aagggtctct 108395822

ctgtgtagcc ctggctgtcc tcctggaact cactctgtag accaggctgg 108395872

cctcgaactc agaaattcac ctgcctctgc ctcccaagtg ctgggattaa 108395922

aggcatgcgc caccaccacc cggctttcat tctggaccct taaaacaaac 108395972

aattttagca ccccgctaca atagcagtca gaattggggt ggtggtgggg 108396022

atcatcagag ctgaaaggga agtggagtca gtcaccgtgt tagttaggaa 108396072

cttaaacaga aacttcaggg cagggaaagc tgcaagtaga aaatggctga 108396122

aattttaagc aacgattcag agctgagaac agtaacccct tgaaaatttg 108396172

taccaatcag atcaatagtt ctagaaattt gtttgtttta acacagtagg 108396222

ttagactctg gttttatgtt tgcataacaa tgatgtattt tagggaaatc 108396272

aatcaatgtt ttaatgacag cccacctgtt gacacaaagt tcatttgtaa 108396322

gattagtcca tcaaaagcct acggactact ttaactctta gccctcctac 108396372

aaaattcttt ctcatacaaa aagctctttc ttttggtatc ctttgcagat 108396422

aaaactatgt ataactaaga tactttactc ttactttaat ttctactgaa 108396472

ctaaagagaa acctatgaaa taaaatattt cagctgtcta ccatgtactc 108396522

atactttatg agtcttataa aaccatttat tgtgtatgtg catctgcatg 108396572

cacgcaggcg cgagcacaca cacacacaca cacacacaca cacacacaca 108396622

tgatgcagga gtgcatgtcc acaaatggtg aagaccagtg aacaacttgt 108396672

aggcatcagt gtctccttcc accacgtggg ttccaaggat caaattcagg 108396722

cggtcaggct tgttagcaaa cacctttacc cactgaacca tctcaccagc 108396772

taaggaaatt aaattttaga gtatccagaa acatgtttcc tcatagagca 108396822

attgatgtat ctggtgtttt aagttattca aaagtaactt ggacatcaca 108396872

tgagtgtcag ttaattccag gtagcataat gtaattctgg aattacattg 108396922

aaagttcgaa ttaattattt atttactttt acctaattta tttattttta 108396972

gctcttaaat ctaaattgct tatgacaact gatatatcag tcctagtgta 108397022

aggctcatct tttgttggtg ttttgctggt ttccaataca tcccaactgc 108397072

agtttctcct ccctccactt ctccagtcct ctcccttctc ccctcctccc 108397122

cagatccact ctgtttctga ggcccaacct ttatcttcct gttaaagaaa 108397172

tgccctatga atgtgttatg tgttgatact caagtgcagt gggcatattt 108397222

gtatatcatg ttcacatact cttgggtcac aattccataa cttatgatct 108397272

taatcatgta acagagaaaa agtcagatct gtattatact taatgttgac 108397322

aacaaaagat atgttagctt taattaggcc aatgatatta tattaccctc 108397372

atttatcaaa gatctacgcg cacagatacc tttacagttt aaggtttcct 108397422

tctgtgtcaa acttagataa agtagtaaac ccaggtgaaa atgtgcagac 108397472

cactctgaag acttcaatta tgtttcacca acaggtttta aatcagccta 108397522

tttatcaaag atttatttaa atcatgtgaa tgaaaaagct tctggattaa 108397572

catattttta gcaagcttaa gtgtactcaa tttatgagga ttcatttatc 108397622

tctaagccaa ttaaaaattc agctcttaaa atccaaggta gaaaaatccc 108397672

aggttcacaa cacagacaca gacacagaca cacagacaca cagacacaca 108397722

gacacacaga cacacacaca cacacacaca cacacacaca gacacacaca 108397772

tacacacaga cagacacaac acacacacac acacacacag agagagagag 108397822

agagagagag agagagagag agagagacta cataatcaga gagagaaata 108397872

aaattcaaag tcttccttta attgggctgg gttgggtgct gggggtaatg 108397922

ttcaatgaga caacataaca ctgtgccctc tgatcatatt ttttatctgt 108397972

aaatcttggt gctagtgggt tacctacagt ttgggtcctg attagatgaa 108398022

gttaagctta agctataaga gatcaagaga aaattgggca cagagtctgt 108398072

gaggagtcct cactcagaaa tgacagcccc agaagtagga ttggtgaact 108398122

ggaattgatg atatcactgt ttgatgctgg gcatggtggc acacagcttt 108398172

aatcccagtc cttaggaggc agaggcatac agatttctgg aagttaaagg 108398222

ttagtctgtt ctacatcatc tgtttcagat ccctaacacc catgacctcc 108398272

acagcaaaca aatagaagct tcttgggagg ctgggtgagt aaatgactat 108398322

aggtaaacgg gtgaggggtg atacaaaggt gagtggatgg atgggtagat 108398372

aggtgagtca gtgagtggac aagtgctact gagcagtacc ggtacctgca 108398422

tgaccaggga gaagcaacaa cataacttta tagccagaac aacagggtac 108398472

aaggctctga aaggctcaaa gaatgatgag gctaagccat ggcacaataa 108398522

aaacctcaga acagaaatca gaccaaagct tatgatctcc agagatttct 108398572

gcccctatct ttaccaaaga catgagagcc cctagttctg tgcacatggt 108398622

ctctcatccc tactcctatt tgtcatctcc taaatgtcac agaaggcttg 108398672

acccaggaca ctatatatcc ctggtccctt ccctacagta gcttctcact 108398722

gggggatggg gatggggttc agtcagtcat ggaaccagac aaggactcca 108398772

tcctgagtac aagacatggt aagattaaat gtagctggac aaggactggg 108398822

gagatagctc agcagttaag agcactggct actcttcgag aggtcctgag 108398872

ttcagttcca agcaaacatg gtggcttctt accatctgta atgggatctg 108398922

atgctctctt ctggtgtgtc tgaagaggat gaaagcatat tcatattcat 108398972

attcataaaa taaataaata aatctttggg ctggagagat ggctcactga 108399022

ctgttcttcc agaggacctg agttcaattc ccagaaacca catgggagct 108399072

aacaaccatc tgtgatgaga tctaataccc tcttctggtg tgtctgaaga 108399122

cagctacagt gtactcacat gcataaaata aatacatctt ttaaaaaaaa 108399172

tgtacccggg cggtggtggc acatgccttt aatcccagca cttgggaggc 108399222

agagtcaggt ggatttctga gttcgaggcc agcctggtct acaaagtgag 108399272

ttccaggaca gccaaagcta catagagaaa ccctgtctcg aaaaacaaaa 108399322

acaaaaaaaa tgtagctgaa catgatgggg gtgtgccccg aaaaatggga 108399372

gagtaaaggc aagggctggc attgtggctc agtgataaag cacttatctg 108399422

atgtgcaaaa ggccttggga ttctgtccca agcacagcaa gagtgcagag 108399472

aggtttgctg ttcacgacag acaaggggaa catggggatt actactaaaa 108399522

cagtgtctct gaaatgagga gctttgtgat tgatgtcatg tatatttctc 108399572

ctggaaagat tagattgcct ggagatgcag cacatccctg agtagatgta 108399622

gctcaagatc atgcctcctc atacactgga tattcaggaa gggtggatgg 108399672

gaatggttct ggggtggaga atatagcatt ataacagagt gataatgaat 108399722

gataacattc actttaggtt gtccaggaag gggctggaag gtttgattgt 108399772

tttaacctgg ttctgtgtgt ccttctctag aggacttcat ctgaaacagt 108399822

tctagaggaa ggcaaaggca ctcctctgga cagcaataaa caaagctgat 108399872

tcaccctata aatttctccc agctactaaa ggaagctagg atgtctgggc 108399922

cacgggctca cgagacccag ggatgtttga ggtagaggag ggatgcatat 108399972

ggttaagggt cagcagaata gaaagggtct tggcctagac aatgggcata 108400022

gagaagtgga ccttgcagaa gtcttcttgg caagaactgt ctcagaagcc 108400072

cactgtattt gtgatacata tgcatctaat aacttggcag tgaagagtta 108400122

aattcagagt ctctagggcc tgagtacctg ccacctttgc tcacttttga 108400172

ggctgtcaga tattctaagc aggaaactat ggaagcaaag aagccctggg 108400222

aagacagtgg aatggacaag aaaggcagga ggaagcctcc agaagctcct 108400272

gaagccacag gatgacaaag aggaagtctg ggagctgcag ggctacatgt 108400322

caatcctcaa tgtggaatgc acatttacat cacatacata gaaaggattc 108400372

gcctctagac gtctagaagt gaggccaaag gctgaaggaa caccatctac 108400422

atgggggact tgtaggacca ggctgaggtt gaagagctac tgagggcatt 108400472

taaaagccca accaaagaca tggcatcctt gagagcattt tatttttttt 108400522

tatttatatt tttttaagat ttacttatta tatgtaagca cactgtagct 108400572

gtcttcagac acaccagaag agggcgtcag atctcattac agatggttgt 108400622

aagccaacat gtggttgctg ggatttgaac tctggacctt cggaagaaca 108400672

ctcgggtgct cttacccact gagccatctc accagttcat tgagagcatt 108400722

ttaaaaccat agctttgatc atagttaatt cccagcaggg gaaagaaata 108400772

tggaaagaga gcaacaaata ggtgagtttc ctgagtggaa agcaggccaa 108400822

gagaaagaga tagggaggtc ccaggctgtt ctacagtttt atttacaaaa 108400872

tgagatttag acaattatag atagaaatgg gcaataaagt gaggtgtcaa 108400922

gtgtgggttg aaatgtggta gaaatcaagc tagtaaggaa agcaaaatga 108400972

gacttgtgtg ccgtgcacat tctctgaagg ccacatctgt agatttgtgg 108401022

gcttcatctg agaatcaaat gtacttcatc taagagtaga cgttcttgac 108401072

cttccaaatg ctgcaaccct ttaatacagt tcttcatgtt gtggtgaact 108401122

ttgggaagcc acatgtgccg ttgcagagtg gcgctggcta ccattggcca 108401172

ctgcccatga ttatgggtaa acaaccaatg tgtgcatgtg cataagaggt 108401222

ttttgccgag tcactgcctg gcccgacgca tattaatgag gtactggtag 108401272

cataaccaat caggtatgga cacgtcactc ctaggcctat ataagcagca 108401322

ccatttctgg gctcagggtc ttttgcctat gcaatcaatg ctctcccaat 108401372

aaacgtgtgc aaagggatcc tattgtggca tcgttcttgc tggcgagacg 108401422

ggcgctcaca gtgaacccaa ccataagatt atgtttgttg ctctttcgta 108401472

actgtaattt tgctactgtt ataaattgta atgtaaatat ctgatattca 108401522

gaatatctca tatgtgacct ctgtgaaaag gtcattcaac tcccaaaggg 108401572

ttcatgaccc acaggttgag aactactgac ctaagagtca gctatatgaa 108401622

cctatgtgat ccatttgagg atcaaattgt gtatctgtat gcatcaaatg 108401672

agagtaagtg taaagatcta tgcatatcat ctgaaggcaa tttcctgctc 108401722

tgtgcaggaa cacttcggta tgcctttgta tagtgcttca agactgctct 108401772

agaagcaacc tgcccatcag gattaccctc tagcactaat agtcagaaga 108401822

taggtgcaaa tcaccaggta tacaagagcc acagtggcaa accatgggga 108401872

gctaccagag acaaagacat ccaggtaaat cccttccttg ggaggatgta 108401922

ccacatgacc aaagcctcgt acccatgatt tagccaaaaa aaaaaaaaat 108401972

tggtgatctt ccgtgacaag atattaatat ttatcccagt aatgttatct 108402022

ctgtcaggtt ttgctgctca tcttcgttct ctgctatcat tcataggtta 108402072

atttatctcc agcttagcac cacatggcag tcaggtggtg gctgcttctc 108402122

caagaaccag gagagaggga aggggaagag ttgattttag tcacctccat 108402172

ccctctgggg agatatccca atcatcctag taggctccta tagctaaaac 108402222

tgtggccttg ctaacctgct accaaggaga cagaatgcca gcaggaaaat 108402272

tttcaagttg ctcctagagc tgagctatgg agaattatga caaaccacgg 108402322

ggctgcaaga tgcatggtct tcagtacata taagaaaatc tcttctaaca 108402372

gtgggacatg tcgaaggtac tgatagtcct cctccatcca ctgaacagga 108402422

gtgggaagga catagaactg ctccaacttt ctgagacagg ccctgaagag 108402472

aggccaaagt cacagccttt cacctcagtc actatgccag ggtgctttag 108402522

tctcatggaa ataaaagaat gaccagaaag caaagagtaa ggcagggtca 108402572

tgtctgttag gagctatccg actctaactt agtatcctga acctagctcg 108402622

aataacctgt tcctttcaga ggtggaacta acttcagaag caggattcct 108402672

tttatatctt ggggttgtag atgtgggagt cctttatgag ccctccttga 108402722

atcctccctg agatcagaga tgggcaaact caccatgtca caaagtctta 108402772

tggtggagca gagttcaagt gtagggttgg tggatagatc ccaagaaata 108402822

ggaaactctc aacttcagct ccccagccaa tttgtcttgg taatagtgtc 108402872

tcataacaag ctaccaaaca caacagagtc catccttcat gcagtcatag 108402922

ttatccagga agagtgtgca gcaggtttgg aggcactgga agaaggagat 108402972

agtcacctgg gctagaagtt ctgagggtag gggcagtggg gcagatttga 108403022

gcatacagga ggccaacttt gcccaaaaga agacaggtgc tctagaactt 108403072

tgtaggggct atttggaaaa cttggaagat ttcttaggaa tcagaggtta 108403122

ggtgagctta gtagaacccc aggaacaggg tgaactctct ctctctgccc 108403172

agagacagtg tcctgaattc agaggtaggg ctacttggca tcacagcact 108403222

agtctaaatg atagccacct aggcatagcc cctctataaa aaacctggaa 108403272

gcaccagact gccaaccata gcaataacct ctctcttccc accacagTGT 108403322 ex2

CCCCACTTCA GCTCATTCGC CGATGAGCTC ACCGACTATG TGACAAAGAA 108403372

CATTTTGTCC ACACCAATCA TGAATGGCAA AGATGTCGTG GCTGTGATCA 108403422

TGGCAGTGAA TAAACTGGAT GGCCCATGCT TCACGAGTGA AGATGAAGAT 108403472

gtgagtgtcc aggagttgtt aaacactcag ctctccccat gggtttgccc 108403522

atgtgtgtgt ctgcgtttgt cccagcatgc ctttgctcat ctgaatgccg 108403572

ttatctctct ttcatgtggt taagtgggcc atgccccttg acaagtctag 108403622

gtctgagttg tgtccctctg ttaatctgaa tgtgtgagct gtgcttatcc 108403672

agaggcgtga gtcacactct cacagagatt cttcaaatct ctctgtgtct 108403722

ctatttatct ctgtgtgtga gctatgcccc agctgttcat ctgtgtgtat 108403772

gtaagtcatg tccctttgtg tgtgatccat gtccttgccc cacaggactg 108403822

aacctttgtg tgcaagtcca cacccatgag tactaagtat agtctaccag 108403872

tattcttgcg tgtgtgtgat acttcctcca gacctttgga gtatgcatct 108403922

cttctgtaca tgtgtcctta aacatctgga tttctggtat gcctcccttc 108403972

tatgcatgtg tccttgaaca tctggatttc tggtatgctt ttttctatac 108404022

atatgtcctt gaacatctgg atttctgact catgctctta taattgctgt 108404072

ttcagGTTTT CACGAAGTAC CTGAATTTTG CTACATTAAA CCTGAAGATC 108404122 ex3

TATCACCTAA GCTACCTCCA CAACTGTGAG ACACGCAGAG GCCAGgtacc 108404172

tgtatgccgt ggctgatcct ttcccagctt tctacattca taggctgttt 108404222

tgtcttcatg aatggccaga cactgtgata tctagtcaaa gcatgactct 108404272

agcctaagag aacctgatgt ttccatccat ttcataggta caaaactgag 108404322

taggagcagg ggtactgtac tcacctaggc aggcaggttg tgagcacatc 108404372

ttgatagttt atgtccatgt ggtaatggtc acttgccaag agatctctgt 108404422

acagatactt caagttcaag gcagaacctg cctccattga tcaaccgaat 108404472

tatggtctat atagcaatgg gagtgcagga caaataccaa gttcagttac 108404522

aacttggccc tgcataaagt acccaggcat gacattgagg ggagggagga 108404572

agagtgaagc tgagagagat gagtgaggta gggaagcaag atctgagaga 108404622

ggaggaccac aaacaggtgc ttcagtcagg caggagccat agaatagaga 108404672

gtccggacta gttagggctg tcaaaggact gtggagatgg caagaccagt 108404722

aaggtaggct gactaggagt caggactgca ggcaagaagc tcagaccatt 108404772

ctgagagagg ggcacatggg ctgagagagg ggaaatgatg acaggctgat 108404822

tggagaggag agagagggaa caatggcttg tcgaaggggg tcacagaggt 108404872

aagtggtcat ttgtagttct caatcttcat tgtactgtca gaagaaaaag 108404922

tagagaaagg agctttaggg atctctagcc ctccaccaaa cccactacat 108404972

gccccatagc ccataacaga catacacacg cacacacaca cacacacaca 108405022

cacacacaca cacacacaca ctcacacact cacacacata catatacata 108405072

caccagaaat aagccaccca caattcaccc caacacacac agcacagaac 108405122

cacaccccat gcaatacact tattgtctgt tccacaccat caaaacgcca 108405172

cacataacct caccatcttc atttcagatg caaattacac atatacatca 108405222

tacatattca cccctgcact gctcccacac agctgcagag gccagctctc 108405272

tcttgttctc cagcctctgt cttcccatct tccatcctaa tgtcttagtc 108405322

agttctcttg ctgtgaagag atactatgac catggcaact cttagaaaag 108405372

aaagcactga gtggggcttg cttaccattt cagaggttta gtccactacc 108405422

atcgtggcag gtaggatggt ggcacacagg cagacatggg gctggacaag 108405472

tagctgagga tttcacatct ggatcctcag gcatcaggag aagagagcca 108405522

tactgggcct ggctgtgata cacttcttcc ttcccaagta gtgccacttc 108405572

ctgatgacta agcattcaaa tatgtgaccc tatgggggcc aactcttact 108405622

caaaccgcaa caccaggttg tgcccaagtg ttctgcactg cccacttcct 108405672

gcctgccatc agttattctc ttgatagaag tggaagctgt cctatctgct 108405722

ctcctacata cataggtcta tcttcagtta aagaaagcca ctgctctccc 108405772

tgcccttcct ccaccctcct gagtggtttc tggaacctca gaattaaccc 108405822

attcccccca tgatgtggtt atttgtgtct atttgaaatt gagtaatgct 108405872

aattcatagc agcggttgct ccagcagaac ccacagttgc ttattctgga 108405922

cattccatcc aatttttgca tgttactgtt tcctgttgtt tttgctttcc 108405972

gtttgttatt tgtggaggtg ggcacactcc atggcctgag tacagagatc 108406022

agagaactac ttaacgaggt tttttttttt ctcttcccat catgtgagtt 108406072

cagcagatca aactcaggtc acatgtaagt gactagtatc cctacccact 108406122

gagtcatctt actagcccca tgatataacg ttatagtctc ttttctgacc 108406172

gttttagatt tacaaagttg agccaaattt gtatagccct caccagcaag 108406222

gagtttctcc attttgacat ctttcattgc tgccacaact aatgaaccag 108406272

caccaactaa caacatcatt aaagactaca cttgattgag atttccttag 108406322

tttttttttc tttttccttc tgtcccagaa tgccatccca aatcccacag 108406372

ttgtcatatc tcctcagacc cctcttgtct gagcaagttt gtcctgtcag 108406422

cttgtttcct gtgacttgaa gagaaacctg gctagcaggg ttcatgtttt 108406472

ttgaattgtt ctgatgtcat tcttgtgatc agacttgggt cactgggttt 108406522

tagagtctta aagtctttcc attgcatcac agtggggtgc caccataggg 108406572

ctctccagaa accaggaaac agtttacatc ccacagtaaa gtcacctgac 108406622

cttctgtcct ttccttgctg aaggaagtca ttgcgcatac catgcaaagg 108406672

aatgggaggt actctcctcc tttggagtgg aacatctata aaatttgtac 108406722

tcatttatta atttgcttaa taattgattc atgtctagat tcatgggtat 108406772

ttgtttactg ctttaggtgg caatccagta ttcactacat tccacaaact 108406822

gttttagccc tgacccttca gctggctcca gtgtctttta acacatttca 108406872

tcactgtagg agtctcaggc ttctccagga tgcttcttgc cctagtccta 108406922

gatcccatca tttctccaag aagccccgat ccatgtacta gaactctcta 108406972

ttgaaagcta agatctgagt attgttatag tctttgttgc tccaatctcc 108407022

tcttacccag caaaggaaga tcacatgtgc taacgtgtgt gcatgcacag 108407072

taggtatatc acatataaca ccatactggt ataaaattaa acataacttc 108407122

tggttgatgt ccctagccct gttctattgc cacgaagatc ctttttggta 108407172

tgtaccctca acagagagac tcctggctct tctcatctac cacccacttg 108407222

ctaaatgtga gaatttttta tcagggattc taatcttcac tcccttgaaa 108407272

aaaatcagag cacagagatt atgccacccc ctttgccagc tgacctaaaa 108407322

ataaatcagc actttctcca tgccccaaag gtgcatagta cagttagatg 108407372

ctcttggtct acattctttt gtgtttagtt ttaaggatgg atgtgtgtgt 108407422

gtgtgtgtgt gtgtgtgtgt gtgtgtgtgt gtgtatgatg tatatgtata 108407472

tggtgtatat gtatatggtg tatatgtata tgtatatgat gtatatgtat 108407522

acatatatat atatatatgg ctggagagat acctcaatag ctaaaaataa 108407572

gcagtgctct ttcagacgtc ctgaattcag cctgcaactg cctataattt 108407622

cacctctaga ggaatacaat gcctctgctt tctgtaggca cctgcattca 108407672

cataaccatg cagttatatg cacatatcta caaacatata tgcccataca 108407722

cgttgcacac acacacacac acacacacac acacacacga tgtggtgcaa 108407772

ggcatataat cccgacactg agatggtaga ttcaagagga tcaccctcaa 108407822

gttccagacc atgcttgtct acacagtaat catagtaaac cctgactagt 108407872

ccagggtttt atagaaattc catgtctcaa acaacaagaa caaaaaggaa 108407922

cacgttcata tgtattttgg atgttagtcc agaagtgcat tttccgtgta 108407972

ttttctccct gagtgtggct tgtcttttca ttctcttaat aatacatatg 108408022

gcaaaacaga agtttggctt ttttcttttt tttttttttt tttttggttt 108408072

ttcgagacag ggtttctctg aatagccctg gctattctgg aactcacttt 108408122

gtagaccagg ctggcctcga actcagaaat ccacctgcct ctgcctccca 108408172

agtgctggga ttaaaggcgt gcaccaccac acccggctga agtttggcat 108408222

ttttataacc ccaatttact atagtcttct tcatagactg ttatacctaa 108408272

aacctcatcc ttaaaccgaa gatcaccaag gttgtctttc aggtcatgtt 108408322

ctaggagtct gagtcttgga ttcttataaa tgcaaatgtt tttatttcct 108408372

cagataaagg gaagagatga gagctgtgcc caagctgtgt gaatacacgg 108408422

ctttggttga ggccagagag ttcctttctc tgcactaact atcccaagcc 108408472

taggcctgaa aacttctagc ctccttacca tctaatctta taaactaact 108408522

gatccaatct ggcttctcct gacttctgac tgaattgctc tgcttggcct 108408572

catactaact ttgacaatat gttctaacct tctggctcct tctcattctc 108408622

tggcttgttc tgccttcacc tgtgtctaac ttgtgtctgt aaactgtcct 108408672

gcccaaactg ctatacacac atcacctcac cactccatct tttttctctc 108408722

ttgttgctct taagcagcct ctctttctct gctgtttcca tgtgagttga 108408772

acacatccta tctttaactc actctgtcaa atccttctgt gattcatcac 108408822

tttatctgtc catcaattaa acatcacttt tgaactggct gcttccttct 108408872

acaaaccaac cttaccttca ttgttaagga tcaaacgtgt gtgtgaattt 108408922

cactactaag ggtgtgtctt aatttcagtc ggattatact gtaatttggg 108408972

atgtatctga atatctttgg atatgacccc ttgccagagc aaccatgttg 108409022

ctggactaaa attcctttaa tgattctgag tttagggctc attattagtt 108409072

tgatgaatac tataaagtct ggctctagat tcacattggc atgtggatat 108409122

caagctttta gtcatttgtg aaaagacaac atttttttct atgaattgtg 108409172

tttccttgtt gttgagggtt aggggttagt tgggtacatt tgtggagatc 108409222

tactttttaa ttttccaggg attttccctg atcagtcact gtagttctag 108409272

agaaaatcgt gaggatgata atgccagtct accaatactg ttttcccttt 108409322

tcagaattgt gttgactatt ctgggtattc tgtctttcca catagacttt 108409372

atagtcaaga tttcaagatt tgtagaatag cttgctggta taacttggat 108409422

ttttctaaat ctatctacca acttggaaaa atattatttt aaaaatcgtg 108409472

agtcttctag ggatgtaagt cagttggtaa gaatgcttcc ctaccatgcc 108409522

tgaaaactgg attcagttcc cagcaccaca tatagcctga catgtgttac 108409572

aagtgtgcaa acctattact tgggaggtgg aggcagaagg atcaggagtt 108409622

caaggtgatc cttgactaaa gagcaagttt gaggctagcc ttggctacat 108409672

gagactgtct cagaaaaaaa tccacaagca cttaaaatct tcatattaat 108409722

tttgatattt tacttctttt ttaaaagatt tacttattta atatgagtac 108409772

accattgctc tcttcagaga caccagaaga gggcatcaga ccccattaca 108409822

gatgtctgtg agccaccatg aggttgctgg gaattgaact cgggacctct 108409872

ggaagagcag tcggtgctct taaccactga gccatctctc cagcaggata 108409922

ttttacttct ttgatcaaac ttttgaagtt ttcatggact ggggaaacag 108409972

ttcacaagca cgagaacctg agtttgttct atgggaccca agtgaaaagc 108410022

caagcatggt accttatttg taacctcagc tctgggaaag tggagacagg 108410072

tgaatcccct agggggactc agtgaccagc tgacttccaa ggtttacctc 108410122

cacacatatg cacacatatg cacatgcacc cgcacacata tgcaaaagat 108410172

tgaagtttct gcatataaat tttatacaca tttcactaga tttataccta 108410222

agtacttcat tttggaacat aatgtaagtg gtgttgtgta ctgatagctt 108410272

catcttccaa ctgttcatca atggaatcaa ggaaagcaat tattatgtta 108410322

atcctattgt ctgtggcttt gctaaaaatc acttactagt ttcagggttt 108410372

tcttattgat tctctggtat tttcatatag ataatcatat cacctgcaaa 108410422

caaagatagc tttattttct ctttttagtg ttttcttttt ctgatctttt 108410472

tttaaaaaag atttatttat ttatttattt atttatttat ttatttattt 108410522

atttattata cataagtaca ctgtagttga cttcagacac accaaaagag 108410572

agcatcagat ctcattatgg gtggttgtga gccatcatgt gattgctggg 108410622

atttgaactc aggaccttca gaagagcagt cattgctttt tgtttatttg 108410672

tttgtttttt gagacagggt ttccttgtgt agccctggct gtcctggaac 108410722

tcactctgaa gaccaggctg tcctggaact cagaaatctc cctgcctctg 108410772

cctcccaagt gctgggatta aaggcgtgcg ccaccactgc ccagctcaat 108410822

cagtcccctc ttttcctggt ctaattggtc aagttaagac tttgtgtaac 108410872

aattataaga gagaagaatc tattttcaat aataattttg aaagatttca 108410922

agtgaacatt ccttcctcat tccaaatctt agagaaaacc acctaatttc 108410972

ttttcttttc agagtgatct tcacaggctg gagagatgcc tcagcagtta 108411022

agagcactgg ctgctcttct agaggtcctg agttcagttc ccagtaatca 108411072

catggtggct cataattata atgagtcttt tggcctgcag gcagaatgct 108411122

atatacataa taaataaata gatcttgaga gagagagaga gagagagaga 108411172

gacagagaca gagacagaga gagagtgatt tcacagccca gtgtggtggt 108411222

gcacaccttt aatcccagaa ctcagaaggc aaagacaggc agacttccgt 108411272

gagactgagg ccagcctggt ctacagagtg agttccagat cagccagggt 108411322

tacacagaga aaccctgtct caaaaatcaa aacaaaacac aacaaagtat 108411372

agggttgctc catggtataa ggagatagca cattggactt ctagaggctg 108411422

aagaatgatg tcatctcttg tggtttcata ggaatttttt ttaatctagt 108411472

taaggaaatt cactgtattt cttgttgtta aggtgctcca tcttgaatta 108411522

atattgaatg ttatcaaata attctgtatc atttatagaa ttatgtaatt 108411572

tttctttagt ttgttcattt taagattata ttagttagct tggggtattg 108411622

gtttgtggta tatttatatt gagctgtggt cttcccatct cacatcaaac 108411672

agctaggtct tcaactgctg ggcttctgtg gttccctcac attagtcttt 108411722

taattgttag tcttactaac aattacatta ttgttacatt acagattggt 108411772

tttatcaatt tcttttgttt tcttagacaa tgatttgcca tgtagttcag 108411822

cttgctatgt agctcttgtc tcccaactgc tagaattata ggtttgtctt 108411872

aacatgcact gtagttttgc cagtgttcag ttaagaagtt ttgtatctag 108411922

agccatgcat gatggtgcat gccttaatca cagcatctag gagggagaag 108411972

caagtggatt ttcatgattt taaggctagt ctgatctaca tagttagttc 108412022

tagggcaatg agggttatgt ggagagaccc tgtctcaaaa taaaaattaa 108412072

aaaaaatgtt tttatatcta tgttaatgac ctacatgaat atataggttt 108412122

tttttccttg tgatgtcttt tttaaaagtt tttaaatttt ttatcattat 108412172

tctttaatct atacttacag tccagttgtt atccccctcc aggtccaccc 108412222

tccaacagtt cctcattcca ttcctcctcc cctgactcca agaggatgtc 108412272

ccccactccc accccctagg cttccccatt ccctagggtc tcaagtccct 108412322

tgagggttag gtgcatcttc tctcactgag gacagacccg gcagtcctct 108412372

gctgtatatg tgtcgggggc ctcatatcag ctggtgtatg ctgcctggtt 108412422

ggtggctcag tgtctgagag atctcagggg tcctggttag ttgagactgc 108412472

tggtcttcct atggggtcac cctcctcctc agcttcttcc agcctttccc 108412522

tgattcagcc acgggggtcc tgtgcttctg tccattggtt gggtgtaaat 108412572

atcttcctct gactctttca gctgcttgtt gggcctcttg gagagcagcc 108412622

atgctagtct cctgtctgta gtaatagtgt caggccttgg agcctcccct 108412672

tgggctggat gtagtgtacg gtgctcacaa gtatggttgc ctgaagaggc 108412722

cagaagtgtt aagatctctt ggatctggag ctacctggtg taggtgctac 108412772

aaacagaact gaagtcttct gcagtagtag aacatgcttt caatcgctga 108412822

gtcatctctc cagtcctagc ctcgtgatgt tttcatctga gtttgttatc 108412872

tgtgctggcc tcggagagga ttttaggcag tattccccat tctgagacac 108412922

agaagaaaat caaactgtct ctctcaaaac acacaatggt tctgtgaaga 108412972

ttttagctac acctcaaaca agcaatcact tctgcatcag acatcagcag 108413022

tgtcctctga ttcagctcaa ctctaatcct atctattcaa agatagtcag 108413072

atcccacaag tgtagggctc agtccaaaga tgacccactt ctggtgccaa 108413122

tgccaaactc tagtctgttg cttgagtttc tgactgatca gctataaggc 108413172

aaatattagt tcattttctt ctgctataat aaattatctg aagctaggca 108413222

tttaacaaat aaaagagatt tagctcatct atgggggagg ttcaagagca 108413272

cagtacaagc ttctgcttga ctatcatgaa gtcctcctgg tattgttgga 108413322

cttaacctgg ggatttggga gaaccatagc attcagccat agtgtttctt 108413372

tggctgtcat tattttttaa tgttgttgct aggttttgtt tgtttgtttg 108413422

tttgtttgtt ttgggggtgg ggtgtttggt ttttgacttt tgccatactg 108413472

acaaaacata ctggcagaca tagcctactg agagtttgtt gtctgagcac 108413522

agaaatacct gaagggggct gggaagatgg ctcagtggtt aagagcactg 108413572

actgttcttc caaaggtcct gagttcaaat cccagcaatc acatgatgac 108413622

tcacaaccat ctgccctctt ctggtgtgtc tgaagatagc tacagcatac 108413672

ttacatataa taaacatttt taaataaatc tttttaagaa gaaaaaggag 108413722

gaggaggaag aggaggagga ggaggaagag gaggaggagg aggaggagga 108413772

ggaggaggag gaagaggagg aggaggagga ggaggaagag gaggaggagg 108413822

aggaggaaga ggaggaagag gaggaggagg agggggaaga ggaggaagag 108413872

gaggaggagg aaagaaatac ctgaagggca atgtaattca tccaagctag 108413922

gagaaaccag tcagcagaga cccagcctga ggcaagggca gttctataaa 108413972

cttgtcatct gaactctatt gagtccagag gtctagacac attctctgcc 108414022

tttgtggaag gtcatgctgg ctctcttctc caaggaatga gaagaaagct 108414072

actagaactc tagcctgcca ttcaaagtct agtcatgtat cagggggctt 108414122

tcctgggatc accctatttt gtaatatccc cttatgtaag tcttaccagg 108414172

gccccaaatc tccaagaaat cagactttag gaagagagga aaagctgccc 108414222

caagactcgg actctaacta gcatttgcag aatccttcct cataccactt 108414272

catgggctat gtttcattta tttttgggaa gctggaaata gagcccagag 108414322

tcttatgcat actaggtaag ttcgccacca ctaagctaca tccctcacat 108414372

tgctttataa aaagaaacct ctgaaggacc tgtgacttag cccaagacac 108414422

gttgcctttc ttgtccctgt gtggtaggag tactggtcac taagcattaa 108414472

aacattttca aactctcggt caccatgatt cccaaatcct gaaggttctg 108414522

agaattagtc ggatcccttt ctgaaaagca tcacagctac actggtcaga 108414572

ggcaataata aattacctga agataagaaa accataggaa accaccacat 108414622

actgatgttc actctgacct gccacttgga tcccagtcag gtccaacaga 108414672

gcagttcttt ctgctcactg caccagtgac cctttgcctc tacctagtgt 108414722

ttgcatggct gaggtgcact gctcacccga aagacatgag gggaggactt 108414772

actgacacaa gataagtaag ggtaaagtca gggacttctc atggtctcat 108414822

gtttccaagt tgtaagcagt gagcattaag gaaggagcca gcctggagta 108414872

cccaccatgg tgtctgtagg cccagagcag ggataaaaga gctgtggttt 108414922

ccacgttctt ccccagtagt tcctcaaaag cagaggccag aacagaaaat 108414972

aaggaagaac aaggaaggtg gggagtctga gtttttattt ttctttcttt 108415022

ctttttcctt tttttccctc ttgaaactgt cttgtttagc tcacattgct 108415072

ctcagtctca ctctgcagta gatgctggcc ttcatcctcc tgcttccagt 108415122

tcccaagtgc caggattata gatatgagcc accattggct ttatcttgat 108415172

tcttagtata atgttttaat gtattagttt acatgactta attttttttt 108415222

ttttttttgg tttttcgaga cagggtttct ctgtatagcc ctggctgtcc 108415272

tagaactcac tttgtagacc aggatggcct tgaactcaga aatccacctc 108415322

cctctgcctc ccaagtgctg ggattaaagg catgtgccac caccgcccag 108415372

ctacatgcct taattttaat aacaatggtg tttttgacac ctgactccca 108415422

ggattcgtag atatttgata acgtgttctt agttcaccag gaagtaaagg 108415472

tctcagtact gatgctagag aacaaaggca tagcagtaag aacagctgag 108415522

agctcatatc ttgattcata agtaagaggg atggaaggga gggcagggga 108415572

gagagacaga gagacagaga gacagcgaga cagagacaga gacagagaat 108415622

aagaacacaa aatcacacaa gtcttttgaa acctcaaagt cctcccctag 108415672

tatcagattt cctccaaata ggccatacat cctaatcttt cccaaacagt 108415722

tctaccaact ggggagaata aatacgttct accaacttag atattaaaat 108415772

atataagact atgtaggtca ttatcatttt taggagactc ggagtcagtc 108415822

ctatgaaaaa acattcttga aatcaggctg cacatgggta agttttgtta 108415872

gagacacaga ttgtcctcct gtttggagtc attcaagaga attgatatca 108415922

tgcagatgtt gctactgagg cccagaaaca ccactcctag acctcttgtt 108415972

gctcagtcct gtgactcata tcaactataa gtttctttct ttctttcttt 108416022

ctttctttct ttctttcttt ctttctttct ttctttcttt ctttctttct 108416072

ttcttccttc cttccttcct tccttccttc cttccttcct tcctttcttt 108416122

ctttctttct ttctttcttt ctttctttct ttctttcgtg caagttgctt 108416172

ttgttttttc tcttctttct ttttttcttt ttcttttttc tttttttttt 108416222

aagtatagaa tagagtttat tcagggcatg ggaaggggag ttaagagggt 108416272

agcagaggca gagaaaggca gagaggaaag agtagagaaa tgggggctgg 108416322

tcatggccac atggagaggg aggaagggaa ttgggagtaa ggggcaagag 108416372

gcaagagaaa ggcaagaacg taagagggta agagaggtct ttcttcctct 108416422

ctctctctct ctctctctct ctctctctct ctcttccttt ccactccaga 108416472

ttttattccc ttcccagtcc accctctgac tgttctacat cccatacctc 108416522

ctccctgccc cctgtctcca caaggatgtc cccacctccc caccccacct 108416572

ccccaccgtc ccatcccatc tgacctctaa actccctggg gcctccagtc 108416622

tcttgagggt taggtgcata ttctctgact gaatccagac cctgaagtcc 108416672

tctgctgtat atgtgttggg ggcctcatat caactggtgt atgctgcctg 108416722

gttgatgatc cagtgtctga gagatctcca gggtccaggt taattgagac 108416772

tgctggtcct ccttatagga tcaccttcct cctcagcttt ttccagcttt 108416822

tccctaattc aaccacaggt gtcagcagct tctctccatt cattgggtgc 108416872

aaatatctgc atctgactct ttcagctgct tgttgggtct ttcaggggac 108416922

agtcatgcta ggtccctttt tgtgagcacc ccatagcctc agtaatagtg 108416972

tcaggcctca aggggacctc cccttgagct ggatcccact ttgggcctgt 108417022

cactcgacct tcttttcctc aggcttctct ccatttccat ccctgtaatt 108417072

ctttcagaca ggaacaatta tgggtcagag ttttgactgt gggatggcaa 108417122

ccccatccct cactcaatga tctttctgct ggaggtgggc tctacaagtt 108417172

ctctctcccc agggtagggc atttcatcta gggtcccttc ctctgagtcc 108417222

taagagtctt tcaacttcca ggtctctggg acattctgga gggtccccca 108417272

acctcttacc tcacaaggtt gcctgttccc attctttctg ctggacctcg 108417322

gggcttcagt ccttttcccc cacccaatac tagatcatgt tcccctctcc 108417372

ccctttccat cccctttccc tcccaggtcc ctccctccct ccccctcttg 108417422

tggttgcttt cttctccctc ccaactggga ctgagatgtc ctcacttggg 108417472

tccttcagtt tgtcgacctt tttgagttct gtggctggag agatggctca 108417522

tcagttaaga gcaatgactg ctcttccaga cttcctcagt tcaatttcca 108417572

gcaacaacac tgtggctcaa aaacatctgt aatgagatcc aaagcaagct 108417622

cagggacaga ggcatgggtg actcaaatac ctgctcattc gatcacatac 108417672

tcaatgctca cactcaacat tcatggactt accctcacac atgtggctac 108417722

acatgcatac ttacaggctt acctgttttt tctcttcaca cacacacaca 108417772

cactgagagt gagagagaga gagagagaga gagagagaga gagagagaga 108417822

gagagagaga gagactgtca gccaggtgtc cttctgaaat ataaaaatgt 108417872

cactgctctt caaagcccat gttcctttct ttttggtagt tctaagactg 108417922

tctaagcaga acagccaaac ttccaaaatt gtggcctcag ctttgtcatg 108417972

gggaagcatg aggtgtataa ggggtgacca gaaatcaccc atgaatgagg 108418022

cagggccaga gtattagagg cataggtggc ctcgggacga aagtaccggg 108418072

atctcaggga tgagcaatgt ggttgaacat ctccagggct ggtcttctga 108418122

tgtgaaggtg aaaatgcagt cctgagcaag attcatgtct cctagaacat 108418172

tgcaggtagt gtggaaagtg tcaaacatgc ccatgtgaac acatgggaga 108418222

cagaggccaa agtgtaaaga ctgatggggg tgcgtctgag caacagaggc 108418272

tgggtcaccc caggaagatg gggactggaa agggttacct cctactagga 108418322

gggctataag gaaattagtt catcaggtcc aaaagaactg gcaggaagaa 108418372

gccaccaagg aaggcccgag agaagggact ctgctataga cccggcacag 108418422

tgtgagtctg aggactgact gggctggtgg tatacattcc agggactcgt 108418472

gtttgagaac catggcctag acctgtgaaa ggagattctt gaacctggtc 108418522

tggtcaagga cctgtgtagc tattaacaaa atagtcactt tcaaattcac 108418572

cctgctgaaa cacaaagggg tctgctgttt atagtgcaag ccttgttact 108418622

ccctgggtta actacagagt gggtgagaat ggttgcttga atctttgact 108418672

tttattattt ttctaaaata cctaaaatag gaattgggca cttaatttta 108418722

agcagcttgt cttgaacctt aaatccctaa aagtaaccat tgtgattaac 108418772

actgatcatt aactccacag tatcctgaat ctcaaaggag cacatttctt 108418822

ttttttttat atatattgat aaaatatggt atctttattc gacttataca 108418872

agcattcttc tttattaata agtcagttta agtggtttca ataatgtaac 108418922

ttacttgaaa ttaaggaaaa tgaaaatatt attaaatctt tgcaaaatca 108418972

cattttcctg atgagaataa aatagaaaag tgtagcataa ctaaaacttc 108419022

ttgagtccca gttgtttaga attataaata ataaaaaaga tatttaagga 108419072

aattaaatac caagctataa atagtagttt aatttattaa aattatggat 108419122

ctttgacttt taagctcaca gtgacttcaa tttctaccca ttggctcatt 108419172

cactgaatga ggccagcact gggtagcaag actcatggtt atggcctaag 108419222

ctagcctgga gaactcacca tccagtgagt ttccagagtt cctctgggtc 108419272

aagacacagg taagactgtg ttcacagtaa gccttgagtt acacaccatc 108419322

agtccgtgtc cttttgggtt cagggccagt tgagcaccct gtaagaatta 108419372

agcttgaact gtttgtggta ccaactctga gtggacccca gaagcttctg 108419422

aggccagatg ggtggggcca caggccaacg tgtgtttcac ccctcagGTG 108419472 ex4

CTCCTCTGGT CAGCCAATAA GGTGTTTGAA GAGCTGACAG ACATCGAAAG 108419522

ACAGTTCCAC AAGGCCTTCT ATACTGTCCG GGCCTATCTA AACTGCGAAC 108419572

GGTATTCAGT GGGCCTCCTG GACATGACCA AGGAGAAGgt gaggtctcaa 108419622

actctcaggc acctcctccc cagtgtgacc cagctcatag actgagtgtt 108419672

cccttctctc ttcttctctt cccagGAGTT CTTTGATGTG TGGCCTGTGC 108419722 ex5

TGATGGGAGA AGCCCAGCCA TACTCAGGCC CACGAACACC TGATGGCCGG 108419772

gtaagtcagg ctgataggtg gcaactaccc acaatcttgc taccctggat 108419822

caaactccat gaagaggatg ggggaatagt gtagccccaa gcacctatct 108419872

actctcatca ggctgcactg ccccagtgag ggtggattct ctagatctgc 108419922

tttaattgcc agagctccta catatattgt gacatgcttc cacatgaatt 108419972

ttagtgtatg tgtggagtat gggccaagtt ggtgtgaagt taaatgggtt 108420022

acaggaaaga gagatctaaa aatctgggtt tgtgatacac atctatgagt 108420072

atgagagaga gagacagagg gagagggaga gagagagaga gagcacatac 108420122

actgtacata tgtaattacc aggtactgta catgtgtcac ctctggatgt 108420172

gcatttttgt gcatggatat gtctgggtgg gtgctgatct cttttcacgt 108420222

aaagaaagac tccattgaga agctgaccag gagtccctac actgatatat 108420272

tttgtttctc agGAAATTGT CTTCTACAAA GTCATCGATT ATATCCTCCA 108420322 ex6

CGGCAAAGAA GACATCAAAG TCATTCCgta agtgctgggg tttgacaagt 108420372

ggctcatacc aggatgggac atccccaaac acaacctcca gagtagccat 108420422

ctttgggatt ttttgtaaag atttattcta tttatatgaa tgtactgtat 108420472

ctgtcttcag acgcaccaga agggagcatt ggatcccatt acagatggtt 108420522

gtgagccacc atgtggttgc tgggaattga attcaggacc tctagaagag 108420572

cagtcactgc ttttaaccgc tgagccatct ctccagccca tctggggcct 108420622

ttatagagag ccagcagtgc cttcccaaaa tagtgaggag gaaatgggca 108420672

gatgaccagg gaagtgacca gcctaaggca cttagatatc tgaaacacat 108420722

atctagagag tcagatgagc tgctctgatg gcctcaccag cagaaactca 108420772

accattccag ccttggcagg tgaaacctcc agaaggtgag gctatcacca 108420822

tatgccaaag aaaagtctag cacagagctc tgccctagag ggacagtagt 108420872

cctatgatta tgtgaccaat gacctcatga aacctgtcaa catcccaaac 108420922

atgctccaag aaggcagtag gattccggta tcatgaatga agaacaggta 108420972

gccagccctg agtctccaga ggccaactga agtcaggagt ctccagtagg 108421022

gaaggagacc ctgcaaaatg aggagttcca acatggcaga atggaaggaa 108421072

gctagctcat cgtgacctcc agtcctcatc agcttcctag cctcatccca 108421122 7-8F 58°C

cctgagctca cagaaaggca taaagtgaac tcatacattg tttcatctaa 108421172

caatgcaagc attcattcct tcgacctctg ttcttttccc acagCACACC 108421222 ex7

**A**

CCCGGCTGAT CACTGGGCCC TGGCCAGTGG CCTTCCAACC TA**C**GTAGCAG 108421272

AAAGTGGCTT Tgtgagtgtc cctctccagg ccttggcctc tactggccag 108421322

tgctatgata tgtgctagcc tgctacctcc tattagcaca tcctgctaga 108421372

tgctgttcac tttgcccagt tagaagctcc tctgttgtag gcaaggttaa 108421422

gtaggttagt gcttgtgttt gtatgttcag ttgctcagag tgaaactgtt 108421472

tctctttctt tccagATCTG TAACATCATG AATGCTTCAG CTGATGAAAT 108421522 ex8

GTTCAACTTT CAGgtaatct gcctacccat ttaatgaaac tgcattttac 108421572

tagaaaaatt ttgtttcaaa aatgatggat tgaattaggt ttttccactt 108421622

gaaacaactt ttggggggct agagagatag gtcagcaggt aaactgtttt 108421672 7-8R 58°C

ctatgatagc atgaggatcc atcacccaca taaataaaac ccaggtacat 108421722

gtacctatca atccaagtgc tgagttaggg ggatgagaag caggcaagac 108421772

aaaaggattc cacaggctta gtagccagct aatctaacca accagtgagc 108421822

tccagtatca gttagaaacc ctgtctcaga aaatagagta gcaagcaacg 108421872

gagaaaaaca tccaacatgc acacacatat atgcacacca atgtatgcaa 108421922

catacacaca taaaacaact tcacttatac ctgtaatccc agcactcaga 108421972

agatgaaggc aggaggtttg ctgcaagtta gcccaaggcc aacattggct 108422022

aaagagtgag attctgtctc gaaaagcaaa tgaaaaatag aaaagaacaa 108422072

tttcatctca gtgactttcc gggaaaccat acaagaaatg tatttgtact 108422122

tccaggttta cagttgagct gattttatgt gggctcagtt agatttaatt 108422172

gatcttggct ggactcaaat gatcttgtct tgtctcagct gaacttatct 108422222

gatctcaact gaccttggct ggactctgca gagcttatct gggctaagtg 108422272

gatcctgtct ggcctcaagt gatcttggct ggtcttagtt gatcttcact 108422322

gggtttatct gggtttagtt ggttttcact gtgtgcagca gaagtcagct 108422372

aatctttgag ctcaatcaat tttggtagga ctcagcggaa ccttatctgt 108422422

tatcatttga ttgaatgtgg tatagttgaa cttttctgtg ctggaactat 108422472

gaagatttag aatttctttg ctctacctgt ccttatctgg ggtttagaca 108422522

aatgtgccta tggccacatg gcactttcct aagggaggtt atgttagtag 108422572

gaacaccact aacagcctct tggaggaact ggttaaaaat taccatccat 108422622

atttcatatt ggaagacaga atgaaccaac tacatagtgt tcaaagtcca 108422672

gtgtaaaata gaaacgtgga tccatttgtt gagcagttag taagttcttt 108422722

tttcagttct tctaataagt attcatgggg actggagccc ttatgtgccc 108422772

atgaagtctg tatgctggga aaatagcctc ctctagccca caaaattacc 108422822

acgcaaagcc cttgaacata aagttcacag acaagtgaaa gtaaagagtc 108422872

agcatgcaac ctattagcat ggtcggagga cagaaaaaaa aaatgaagtc 108422922

taaccctgcc cctcccccac cctggtcacc aagtccttac cagagcacac 108422972

ctggaacagc ctagttcttt ccctactccc tgcagGAGGG GCCCCTGGAT 108423022 ex9

GATTCAGGAT GGGTGATCAA GAATGTCCTC TCTATGCCCA TTGTCAACAA 108423072

GAAAGAGGAG ATTGTGGGGG TGGCAACCTT CTACAACAGG AAAGATGGAA 108423122

AGCCCTTCGA TGATCAAGAT GAAGTCCTTA TGGAGgtaaa tatctagtat 108423172

catacatgga caaaagggtc catctatcat gtcgtgggat atcagtgagg 108423222

actgtccctc tccacacata tttggaccct gattagcact acatcaggga 108423272

gaaggggaca aagatagttc ccaaagttct atcttgggag acccattcat 108423322

agacatccac tgtactgtag cagtctggga aactggcctc tgagacaccg 108423372

gtataaaacg tagttaaaac tcaccaaagt gcatggctaa tggggcgaca 108423422

ctcctgtcta cctccagTCT CTGACACAGT TCCTGGGGTG GTCAGTGCTG 108423472 ex10

AACACAGACA CCTATGACAA GATGAACAAG CTGGAGAACC GTAAGGACAT 108423522

CGCCCAGGAC ATGGTTCTGT ACCATGTGAG ATGTGACAAA GATGAAATCC 108423572

AGGAAATCCT Ggtactgttc ttggtatttt atggggcccc tctgactggg 108423622

ggaggcaaag aactcagtga taaggatata tgtatggggc ccaatggcta 108423672

ggtctaaccc taacactaca cactgtgcaa cccagacact gtccctgtgc 108423722

ctcatcctaa gcagtgagta ggtgtgaaca gaaccttcct agaccatctc 108423772

attattccat ctgtaatcca tatgtgtcac taaggacaac acagggaaag 108423822

gaataaacgt cagggtcccc aggaacttac cacttaggtc tggaattgag 108423872

gatgtctgag cccaagggag actgctcttc ttcagcgatt atcctgtgtt 108423922

caacatgttc tgtgatggta taccaccatt ctgttacatt cacagggtaa 108423972

gggtgatgtc actgtcacag gagtaaagga gataccacca ctgacacact 108424022

tatagatgga catagatggc atgataccac cattatgtca cactggtgtg 108424072

aaggttaata gttactcagt tatagtcata gggttaaggt gatcctaagg 108424122

tgatcctgtc ccttgtgggg gaaaggtaat gaggtcacta tgttactgtc 108424172

attcctggga tgagagtgat attgatggct atcatatggg gtgtaggtga 108424222

tgctgttact ctgttatata gggtgaagtg acactatcac tcagtcccac 108424272

aactcacctg ttgctctctg ttagCCAACA AGGGATCGCC TTGGGAAAGA 108424322 ex11

GCCTGCTGAC TGTGAGGAGG ATGAGCTGGG GAAAATCTTG gtgagaatct 108424372

gcttccagtg gacacaagct gctctcaaga gtgtcaggag agaggcctgg 108424422

cacacacagc atcctctcat gatgttagtg ctacccatgt aggcatgtca 108424472

acactcccca gcagagagga caatattggc tcgtaaagtg tccagctgga 108424522

gtttacttga tatctgtcct gctgagtgtc tctgctgtct tgtctcctct 108424572

gtttcccaga actagccatg ttttagtttc tgcatgcaca atttaggccc 108424622

actgtccatc tgtgctgcct ccctgtagga gcatctaggt actacagtcc 108424672

ctattttaga tatatctatg ttcatgtgtg tgcatacttc tgtggacaca 108424722

catgtgtgta tagacacaca gaaacacctg tttgtgtgtc tcccagagaa 108424772

cacccgtaca tcaacttgac aattgtgggg cagctacctt tttctgtcac 108424822

ctgtgtgatt tatcagaatc accagagctg tttgttttac tgaacgagct 108424872

ttggagttta ttaataccaa caagttctaa acaccaatag ctctgtgtgt 108424922

cgtgagtgtg cacatatgtg cccatatgtc catatgggta ggtctaaatc 108424972

catgagtgta cctctgtaac ttttcaagta tatatcttat atgtttgctt 108425022

ttctaacatc actgtcacca agccaggcaa gcttctcact gtcaggttac 108425072

ctggtgcaag gaaacagtag gataagataa acagtggcct agctataaat 108425122

ttgagatata cctgggtgtc tgcttggaca atggttcaga gaaaagaaag 108425172

gttaagaagc agtaagggtg tggtaggcct gcctctctca gcccacaatc 108425222

tctcttacag AAGGAAGAAC TTCCAGGGCC GACCAAGTTT GACATCTATG 108425272 ex12

AGTTCCACTT CTCTGATCTG GAGTGTACAG AGCTGGAGCT AGTCAAATGT 108425322

GGCATCCAGA TGTACTATGA GCTGGGTGTA GTCCGAAAGT TCCAGATTCC 108425372

CCAGGAGgtg aggagatatg gttggaggtt ccagaacaag aggaagtaga 108425422

ggggtggtaa aggttacagg tcccccaaga ggtgaagtgt atatgtatgt 108425472

aacagaggga gggtgtccac aagtcccaca gagaatctag tcaaacatgt 108425522

ccatcacagt gaagtatact tggcaaatat atctagaata aatgattgga 108425572

tggacggaaa gtttgttaac taggtgtcca aatgacattt ctattaggac 108425622

atcacaggga aataagatcg ttggtgggga actgtagcaa ctgctcacca 108425672

ggacagaaat cacatagtat cttctagctt tgcctctcag ggatcaatat 108425722

ttggaggaag ggaagggctc agagccccac catcctgctc ttaccatggg 108425772

tttctcactc tgtcctgagt ccacctcctt acctgtgctc ccacaacaca 108425822

aatgaagaat gaataatctc tcatgctcct cagaaaacag aagcaacagg 108425872

ctgatatttc tgaaagaaat tttactaaat gctaagggaa acagacagaa 108425922

agaaagccat tgggataaaa aatatttcca aataattatt tgatcctaaa 108425972

tatcataatg gaaggggtaa agggttctct tagcctgcat cctcttgttg 108426022

aagaaaatgt tgaggcttcc cacctttctg ctcacaggcc acatgtgaat 108426072

caaacactca ggccttaaac caatgggttt ttttgttgtt gttgttgttg 108426122

ttgggttggt tatttagttg gttttggttt ttgctttctc aaaagatctc 108426172

acatgccagg ggtcctcttt ccattgtgag tctgaacctt gggaagagag 108426222

aggcatcaaa ttcccaaggg ctgttctttg ttctggtgtg agagcccttc 108426272

ccaaacagga aggggccagt gagaacaagg aacaagggct ctgagaccaa 108426322

caagatagaa gtggagaaat aggtataaca gactctaatg caagcagtat 108426372

gagaggcttg gataggctct gatatggtgc tgtgtaggct catatgtgga 108426422

tctcagaacc cacatgtact ctgctcccca gGTCTTGGTG CGCTTTCTAT 108426472 ex13

TCTCTGTCAG CAAAGCCTAT CGAAGAATCA CCTACCACAA CTGGCGCCAC 108426522

GGCTTCAATG TAGCCCAGAC CATGTTTACC CTACTCATGg tacgtatgta 108426572

aattggatgg gctagatgaa tcagagggct ggggcaagga ccacagctaa 108426622

ctatcttctg gcccaaggat gccaattgtg tgtatccagt cctagcaatg 108426672

agtggaaggg acctgggtgg gcaaagggat gggtttggga caccccacat 108426722

cccctttaac cactgccttc tggagccaga gagctgggcc agtgacagga 108426772

ctcaggactg gatctattcc cacagACAGG CAAACTGAAG AGCTATTACA 108426822 ex14

CTGACCTAGA GGCCTTCGCC ATGGTTACAG CTGGCTTGTG CCACGACATC 108426872

GACCACCGTG GCACCAACAA CCTGTACCAG ATGAAgtagg ctgtggggag 108426922

ggcaggcttg tggtgggcat ggtaaaccat aaatcacgcg tccttagggt 108426972

gagtctttgc agtctggcaa tcagtactga gatggaccac cttgaagatg 108427022

cagtgccaca tgggtcagac ctcatcctac tgccttctaa cattgctttt 108427072

gttgtgttcc cctgttaaat aaaataaaca acagATCGCA GAATCCTCTA 108427122 ex15

GCCAAGTTAC ATGGCTCCTC AATTCTGGAA AGGCACCACC TGGAATTTGG 108427172

GAAGTTTCTG TTGGCAGAGG AGgtttgttc ctgatttggt tatgagttgt 108427222

ttctcctctg ggctgtggta gacaagcagg aggtcctggg cagcatggat 108427272

ttatcagtgt gaggtgctaa aggacaggct cacatctaga gatgttccca 108427322

catggggcag aattgcagtc atggaggcag tagctaggaa tggccactag 108427372

agggtacaac agatcatgga aatgggcaaa ggtatctgac ccaggacact 108427422

gacagagggt agacaccaag ggaaggcagg gatggccata agactgatct 108427472

tcacataagt gaaggcaagg ttcgagtgag gggattagac agagtaggcc 108427522

ctgagagtca aaaacaagag taccacagac aacctctgcc acaacccaac 108427572

accaacttat ggtccccaca gAGCCTGAAC ATCTATCAGA ACCTGAACCG 108427622 ex16

GCGGCAGCAT GAACATGTGA TCCACCTCAT GGACATTGCC ATCATTGCCA 108427672

CCGACCTGGC CCTCTACTTC AAgtaagaat gcatcagagg caggaggtgt 108427722

gggcagagcc ctcagtagac atagataacc atggccagtc tgtcacctac 108427772

tcctcccctc cttcttgggg tcagGAAGAG AACAATGTTC CAGAAGATTG 108427822 ex17

TGGATGAGTC TAAGAACTAT GAAGATAAGA AGAGTTGGGT TGAGTACTTA 108427872

TCCTTAGAGA CCACACGAAA GGAGATAGTC ATgtaagtag caggtccact 108427922

gctggccaat tagccctgca gacctcccta atcccaggga ccggagacgg 108427972

cacaagggtt cataggcctc accactgaat ccagaatctt ccttagcacc 108428022

cctgaccacc acttgcttca agctcagctt tccttcttca cctacaaggt 108428072

ggtgacttgg gaccagcacc tttcctagga ctgtcccaga gagctgctga 108428122

gaaaatgttt actctctgct cagaaggaca ggtagtctgc tgtctgtctg 108428172

tccacagGGC CATGATGATG ACTGCATGTG ACCTGTCTGC TATCACCAAA 108428222 ex18

CCCTGGGAAG TCCAGAGCAA Ggtcaggact gatgaccatc tggacccaga 108428272

atagcccttc gcaggccatc ctgggtctgt acctagggag ctacagggaa 108428322

atggtgttgg tttttgaagt catccctggg tacggcagtg agaggcgcca 108428372

tatgaacact gcctgtagga ccttgagggg agggcctact gctcctgaac 108428422

atggagcccc actataactt ttgtgtgtgc ttagGTTGCT CTTCTGGTGG 108428472 ex19

CTGCTGAGTT CTGGGAACAG GGGGACTTGG AGAGGACTGT CTTGGATCAA 108428522

CAGCCCATTg tgagtgttgc ttccagaata ttctatcttg gcatatgtat 108428572

tagaatgctt ggtagcgctc cagaccccag gaatttaagg tcccggagtc 108428622

ttagccttga tttgtgtggc cctttggcat gactatgggt aattttgcat 108428672

cttgcagaat agaaatcaca aaaaattgtt agattggggt tgtagctcag 108428722

caggagcacc tagggtctat gtggctgcag agtacatcca ttagacaggc 108428772

cagtccaggg aggtcaagtc agagaagggc aagcctaggg aaaggcaggg 108428822

agtagtggtg gtagtccatg gcaagataga caatgaggta gttacagcta 108428872

gggttagggc ccaccactaa caccacagat ggcatctaca tacatgggaa 108428922

ctgggaacta caaagggttg ttctgaccac ttatcttcta agccagctct 108428972

ggctagtgtg aagaactgtt tggagcaggc tttaggcaag gcatagacta 108429022

agcaagaaat aggtctttgc agcaaacaaa gtctcatagg gagagacagc 108429072

acccatgtca tctgcctgtg ttttctttac aagCCCATGA TGGACCGGAA 108429122 ex20

CAAAGCAGCT GAGCTCCCTA AGCTGCAAGT TGGTTTCATT GACTTCGTAT 108429172

GTACTTTCGT ATACAAGgtg cgcagcagag gtattccatg ttggctgggg 108429222

tggactggct cagagtgggt tgggaatgga ctgaatccag cagggatcac 108429272

acagggacat ttgtgtgatc ttcaccctta tttgatgggg gaaaccacaa 108429322

cacccacctc aacctgccct gctaattgtc ccaaaactta tgactgttcc 108429372

ctcagaagca ctaagtgtat ttcctaaaat gtccttactc accttgaggt 108429422

ctcaccttaa atatctctgc ctcagagggc ctctttaacc acaatgggca 108429472

caccatcttc aggaaaatgg ctacaacttg tctactaggt atctgtgaag 108429522

gtgataccta gttttttgtt gttttttgtt ttttgttttt ttttgttttt 108429572

tgtttttttg ttttccagac agggcttctc tgtatagccc tggctgttct 108429622

ggaactcact ctgtagacca ggctggcctt gaactaagaa attcgcctgc 108429672

ctctgcctcc caagtgctgg gattaaaggc gtgcgccacc actacctggc 108429722

aagcctgagt cttatctgtc tccaatggca taccccaata caacctgaag 108429772

aacatgatag cagccatgtt cccactagct ggtcaggcac cgatgaactg 108429822

ccaagtcgtt aagttctgct atttgatcat agagtgtaag acagggactt 108429872

gaagagactc acaagatagt gtcctcatgt gatgtacatc acacccaagc 108429922

tccacaccac tctagtactc agtggtgaca gaccccacat tgtaaggtag 108429972

atgctatgtg acttcaagtg aagtcaatcg taagcagcca aggaggaagg 108430022

aagaggtggt cactgtggac cccactgttc ctctgcccat tgacatcacc 108430072

tgaaaacaga tgtagggctt gttagagctt ccaatcctcc tgcaggtgac 108430122

attctctatt cttgggatat tcaacctgct agtactgtat cataaatact 108430172

tatacccagc agccccatct cacaaaagtt cctatcctgt ccccctcctc 108430222

aggaattttc tgggaaggag acacctcagt ggttctctcc tatgcgggtt 108430272

aactatagat ggcattgcct ggtgcgagca ccaagtgaaa tgagctaagg 108430322

atcaggaaac caagcttatg ctgggcctca tttcactgtc cttatgactt 108430372

ctaagataaa tatgccacag ataccagaat cctataacca agatctatag 108430422

ctccacgatt ctagagcatt ctcagtccaa tgagtctggt ggtagggaag 108430472

cctgaggcct ttaagagcta ccatttgagc cctccgggat cagtgaaaca 108430522

gcatggtgac ttctcagttc cctctagGAA TTTTCTCGAT TTCACGAAGA 108430572 ex21

GATCCTGCCC ATGTTTGACC GACTGCAAAA TAACAGAAAG GAGTGGAAAG 108430622

CACTAGCTGA CGAGTATGAG GCCAAAGTCA AGGCCCTGGA GGAAGAAAAG 108430672

AAGAAGGAAG AAGACAGAGT CGCAGCCAAG AAAGgtctgg gcccctcaga 108430722

gaactgggat gcgctcatag ttaaccccta agtcaatgct gtaagatggc 108430772

agagaaccac caagcaagca gcaaggcagc taattcagac actccgacag 108430822

ctgagacaga ggctttggag ccaaggaatg tcccattcat ctatcctgtc 108430872

ccccatggag catggctgtt catgctatgt ggacaacaga ccagactgag 108430922

atgttctctc actgagacaa gaatactagt atatccattc ttatacaggg 108430972

gaagtaagct cactcaagtc ctatcagcct cttgttccac taagaagttc 108431022

cttgccaggt gtggtagcat atgcctgtaa tcacataccc cctccccaag 108431072

ctatcctcag agtcaagaga gaaaacctga accaaacaac aaaaaggcaa 108431122

agacactgag ctaagcaaga gttttaagca ggcacagtgc ttaccctata 108431172

tcatgtgaat ctgttgaaca agctgatgcc cccaggatca gaagcccagt 108431222

ggcactctct tatcttttcc agggtttgtt taccctggac ctggggttct 108431272

ccagggccac tgtaagagca cattgaagag cacagcttct gggtcatagg 108431322

aggtcaggac tgaaagtaat cagggcatag tgaccgaaga ggctcaagtg 108431372

ctaagcaagg ctcagcataa tcggcttggg ttggctacag acctagttca 108431422

gcccccgcag ggagaaccta cagggtcaga gggcactggt tatagaagac 108431472

gaaggctcac cctcttatct tcctttgcag TGGGCACAGA AGTTTGCAAT 108431522 ex22

GGTGGTCCAG CACCCAAGTC CTCGACCTGC TGTATCCTA**T AA**GCCGTGTT 108431572

TCATGGCTTT GGGTCCCTCC TGCCACTCAC CCACTGCATA CGGACTCTGC 108431622

CTATAGCAAT TTGGTATAAG CCATTAGGAA CAGGAAAAAA AAAAAGATCG 108431672

AATGTAACTT TGGATATTTT GAAATTTTAA AGGTGTTTTT ACAAATTAAA 108431722

AACAACTATG AATAAGTTAA Atggtttggc ccataatatt tatctggttt 108431772

gataaaagat cccacagaga tctttataaa ggatctgtgg aatgtgtctg 108431822

gaatcagagc tctactattg t

1 atgagcctcagtgaggaacaggtacgcagcttcctggatgggaac

M S L S E E Q V R S F L D G N

46 cccacgtttgcccatcaatactttgggaagaagttgagccctgaa

P T F A H Q Y F G K K L S P E

91 aatgtggcaggggcctgtgaagatggttggctggcggactgtggc

N V A G A C E D G W L A D C G

136 agcctgcgagagctgtgccaggtggaagagagtgcagcacttttt

S L R E L C Q V E E S A A L F

181 gaactggtgcaggacatgcaggagagtgtcaatatggaacgtgtg

E L V Q D M Q E S V N M E R V

226 gtcttcaagatcctgcggcgcctctgcaccatcctgcatgccgac

V F K I L R R L C T I L H A D

271 cgctgcagcctctttatgtaccgccagcgcaatggcatagctgaa

R C S L F M Y R Q R N G I A E

316 cttgctacgcggctcttcagcgtgcagcctgacagccttctggag

L A T R L F S V Q P D S L L E

361 gattgcctggtgccccctgactctgagatcgtcttccccctggac

D C L V P P D S E I V F P L D

406 attgggattgtgggccatgtggctcagaccaagaagatgataaac

I G I V G H V A Q T K K M I N

451 gtgcaggatgtggcagagtgtccccacttcagctcattcgccgat

V Q D V A E C P H F S S F A D

496 gagctcaccgactatgtgacaaagaacattttgtccacaccaatc

E L T D Y V T K N I L S T P I

541 atgaatggcaaagatgtcgtggctgtgatcatggcagtgaataaa

M N G K D V V A V I M A V N K

586 ctggatggcccatgcttcacgagtgaagatgaagatgttttcacg

L D G P C F T S E D E D V F T

631 aagtacctgaattttgctacattaaacctgaagatctatcaccta

K Y L N F A T L N L K I Y H L

676 agctacctccacaactgtgagacacgcagaggccaggtgctcctc

S Y L H N C E T R R G Q V L L

721 tggtcagccaataaggtgtttgaagagctgacagacatcgaaaga

W S A N K V F E E L T D I E R

766 cagttccacaaggccttctatactgtccgggcctatctaaactgc

Q F H K A F Y T V R A Y L N C

811 gaacggtattcagtgggcctcctggacatgaccaaggagaaggag

E R Y S V G L L D M T K E K E

856 ttctttgatgtgtggcctgtgctgatgggagaagcccagccatac

F F D V W P V L M G E A Q P Y

901 tcaggcccacgaacacctgatggccgggaaattgtcttctacaaa

S G P R T P D G R E I V F Y K

946 gtcatcgattatatcctccacggcaaagaagacatcaaagtcatt

V I D Y I L H G K E D I K V I

991 cccacacccccggctgatcactgggccctggccagtggccttcca

P T P P A D H W A L A S G L P

X

1036 acctacgtagcagaaagtggctttatctgtaacatcatgaatgct

T Y V A E S G F I C N I M N A

1081 tcagctgatgaaatgttcaactttcaggaggggcccctggatgat

S A D E M F N F Q E G P L D D

1126 tcaggatgggtgatcaagaatgtcctctctatgcccattgtcaac

S G W V I K N V L S M P I V N

1171 aagaaagaggagattgtgggggtggcaaccttctacaacaggaaa

K K E E I V G V A T F Y N R K

1216 gatggaaagcccttcgatgatcaagatgaagtccttatggagtct

D G K P F D D Q D E V L M E S

1261 ctgacacagttcctggggtggtcagtgctgaacacagacacctat

L T Q F L G W S V L N T D T Y

1306 gacaagatgaacaagctggagaaccgtaaggacatcgcccaggac

D K M N K L E N R K D I A Q D

1351 atggttctgtaccatgtgagatgtgacaaagatgaaatccaggaa

M V L Y H V R C D K D E I Q E

1396 atcctgccaacaagggatcgccttgggaaagagcctgctgactgt

I L P T R D R L G K E P A D C

1441 gaggaggatgagctggggaaaatcttgaaggaagaacttccaggg

E E D E L G K I L K E E L P G

1486 ccgaccaagtttgacatctatgagttccacttctctgatctggag

P T K F D I Y E F H F S D L E

1531 tgtacagagctggagctagtcaaatgtggcatccagatgtactat

C T E L E L V K C G I Q M Y Y

1576 gagctgggtgtagtccgaaagttccagattccccaggaggtcttg

E L G V V R K F Q I P Q E V L

1621 gtgcgctttctattctctgtcagcaaagcctatcgaagaatcacc

V R F L F S V S K A Y R R I T

1666 taccacaactggcgccacggcttcaatgtagcccagaccatgttt

Y H N W R H G F N V A Q T M F

1711 accctactcatgacaggcaaactgaagagctattacactgaccta

T L L M T G K L K S Y Y T D L

1756 gaggccttcgccatggttacagctggcttgtgccacgacatcgac

E A F A M V T A G L C H D I D

1801 caccgtggcaccaacaacctgtaccagatgaaatcgcagaatcct

H R G T N N L Y Q M K S Q N P

1846 ctagccaagttacatggctcctcaattctggaaaggcaccacctg

L A K L H G S S I L E R H H L

1891 gaatttgggaagtttctgttggcagaggagagcctgaacatctat

E F G K F L L A E E S L N I Y

1936 cagaacctgaaccggcggcagcatgaacatgtgatccacctcatg

Q N L N R R Q H E H V I H L M

1981 gacattgccatcattgccaccgacctggccctctacttcaagaag

D I A I I A T D L A L Y F K K

2026 agaacaatgttccagaagattgtggatgagtctaagaactatgaa

R T M F Q K I V D E S K N Y E

2071 gataagaagagttgggttgagtacttatccttagagaccacacga

D K K S W V E Y L S L E T T R

2116 aaggagatagtcatggccatgatgatgactgcatgtgacctgtct

K E I V M A M M M T A C D L S

2161 gctatcaccaaaccctgggaagtccagagcaaggttgctcttctg

A I T K P W E V Q S K V A L L

2206 gtggctgctgagttctgggaacagggggacttggagaggactgtc

V A A E F W E Q G D L E R T V

2251 ttggatcaacagcccattcccatgatggaccggaacaaagcagct

L D Q Q P I P M M D R N K A A

2296 gagctccctaagctgcaagttggtttcattgacttcgtatgtact

E L P K L Q V G F I D F V C T

2341 ttcgtatacaaggaattttctcgatttcacgaagagatcctgccc

F V Y K E F S R F H E E I L P

2386 atgtttgaccgactgcaaaataacagaaaggagtggaaagcacta

M F D R L Q N N R K E W K A L

2431 gctgacgagtatgaggccaaagtcaaggccctggaggaagaaaag

A D E Y E A K V K A L E E E K

2476 aagaaggaagaagacagagtcgcagccaagaaagtgggcacagaa

K K E E D R V A A K K V G T E

2521 gtttgcaatggtggtccagcacccaagtcctcgacctgctgtatc

V C N G G P A P K S S T C C I

2566 ctataa 2571

L *

#### cDNA

Mouse ID_964_Dkk3 :Cre ;DCCfl/fl

CAGSCTMAGAAGGCATAAAGTGAACTMTACATTTTTTMTCTAACAATGCAAGCATTCATTCCTTCGACCTCTGTTCTTTTCCCACAGCACACCCCCGGCTGATCACTGGGCCCTGGCCAGTGGCCTTCCAACCTA**C**GTAGCAGAAAGTGGCTTTGTGAGTGTCCCTCTCCAGGCCTTGGCCTCTACTGGCCAGTGCTATGATATGTGCTAGCCTGCTACCTCCTATTAGCACATCCTGCTAGATGCTGTTCACTTTGCCCAGTTAGAAGCTCCTCTGTTGTAGGCAAGGTTAAGTAGGTTAGTGCTTGTGTTTGTATGTTCAGTTGCTCAGAGTGAAACTGTTTCTCTTTCTTTCCAGATCTGTAACATCATGAATGCTTCAGCTGATGAAATGTTCAACTTTCAGGTAATCTGCCTACCCATTTAATGAAACTGCATTTTACTAGAAAAATTTTGTTTCAAAAATGATGGATTGAATTAGGTTTTTCCACTTGAAACAACTTTTGGGGGGCTAGAGAGATAGGTCAGCAGA

Mouse ID_3756_Dkk3 :Cre ;DCCfl/fl

CCCTGRCCTMAGAAGGCATAAAGTGAACTMTACATTGTTTMTCTAACAATGCAAGCATTCATTCCTTCGACCTCTGTTCTTTTCCCACAGCACACCCCCGGCTGATCACTGGGCCCTGGCCAGTGGCCTTCCAACCTA**C**GTAGCAGAAAGTGGCTTTGTGAGTGTCCCTCTCCAGGCCTTGGCCTCTACTGGCCAGTGCTATGATATGTGCTAGCCTGCTACCTCCTATTAGCACATCCTGCTAGATGCTGTTCACTTTGCCCAGTTAGAAGCTCCTCTGTTGTAGGCAAGGTTAAGTAGGTTAGTGCTTGTGTTTGTATGTTCAGTTGCTCAGAGTGAAACTGTTTCTCTTTCTTTCCAGATCTGTAACATCATGAATGCTTCAGCTGATGAAATGTTCAACTTTCAGGTAATCTGCCTACCCATTTAATGAAACTGCATTTTACTAGAAAAATTTTGTTTCAAAAATGATGGATTGAATTAGGTTTTTCCACTTGAAACAACTTTTGGGGGGCTAGAGAGATAGGTCAGCAG

Mouse ID_961_Dkk3 :Cre ;DCCfl/fl

TWWKKMTWMAWRAAAGGCATAWWGTGAACTMTAYATTKTTTTMTCTAACAMTGCAAGCATTCATTCCTTCGACCTCTGTTCTTTTCCCACAGCACACCCCCGGCTGATCACTGGGCCCTGGCCAGTGGCCTTCCAACCTA**C**GTAGCARAAAGTGGCTTTGTGAGTGTCCCTCTCCAGGCCTTGGCCTCTACTGGCCAGTGCTATGATATGTGCTAGCCTGCTACCTCCTATTACACATCCTGCTAGATGCTGTTCACTTTGCCCAGTTAAAGCTCCTCTGTTGTAGCAAGGTTAAGTAGGTTAGTGCTTGTGTTTGTATGTTCAGTTGCTCAGAGTGAAACTGTTTCTCTTTCTTTCCAGATCTGTAACATCATGAATGCTTCAGCTGATGAAATGTTCAACTTTCAGGTAATCTGCCTACCCATTTAATGAAACTGCATTTTACTAWAAAAATTTTGTTTCAAAAATGATGGATTGAATTAWGTTTTTCCACTTGAAACAACTTTTGGGGGGCTAWAAGAGATAKGTCAWCAG

Mouse ID_138_DCC Kanga/-

AKGTMGGAMTMAGCARGCATAAAGTGAACTMTACATTGAATMTCTAACAATGCAAGCATTCATTCCTTCGACCTCTGTTCTTTTCCCACAGCACACCCCCGGCTGATCACTGGGCCCTGGCCAGTGGCCTTCCAACCTA**C**GTAGCAGAAAGTGGCTTTGTGAGTGTCCCTCTCCAGGCCTTGGCCTCTACTGGCCAGTGCTATGATATGTGCTAGCCTGCTACCTCCTATTAGCACATCCTGCTAGATGCTGTTCACTTTGCCCAGTTAGAAGCTCCTCTGTTGTAGGCAAGGTTAAGTAGGTTAGTGCTTGTGTTTGTATGTTCAGTTGCTCAGAGTGAAACTGTTTCTCTTTCTTTCCAGATCTGTAACATCATGAATGCTTCAGCTGATGAAATGTTCAACTTTCAGGTAATCTGCCTACCCATTTAATGAAACTGCATTTTACTAGAAAAATTTTGTTTCAAAAATGATGGATTGAATTAGGTTTTTCCACTTGAAACAACTTTTGGGGGGCTAGAGAGATAGGTCAGCAG

Mouse ID_142_DCC Kanga/-

CCGGCCTMAGCARGCATAAAGTGAACTMTACATRGAATMTCTAACAMTGCAAGCATTCATTCCTTCGACCTCTGTTCTTTTCCCACAGCACACCCCCGGCTGATCACTGGGCCCTGGCCAGTGGCCTTCCAACCTA**C**GTAGCAGAAAGTGGCTTTGTGAGTGTCCCTCTCCAGGCCTTGGCCTCTACTGGCCAGTGCTATGATATGTGCTAGCCTGCTACCTCCTATTAGCACATCCTGCTAGATGCTGTTCACTTTGCCCAGTTAGAAGCTCCTCTGTTGTAGGCAAGGTTAAGTAGGTTAGTGCTTGTGTTTGTATGTTCAGTTGCTCAGAGTGAAACTGTTTCTCTTTCTTTCCAGATCTGTAACATCATGAATGCTTCAGCTGATGAAATGTTCAACTTTCAGGTAATCTGCCTACCCATTTAATGAAACTGCATTTTACTAGAAAAATTTTGTTTCAAAAATGATGGATTGAATTAGGTTTTTCCACTTGAAACAACTTTTGGGGGGCTAGAGAGATAGGTCAGCAGA

Mouse ID_145_DCC Kanga/-

AGARAAASAMTAGWGCARGCAYAAAGTGAACTMTACRGGGAAAAAMWCTAACAATGCAAGCATTCATTCCTTCGACCAMAGTTCTTTTCCCACAGCACACCCCCGGCTGATCACTGGGCCCTGGCCAGTGGCCTTCCAACCTA**C**GTAGCAGAAAGTGGCTTTGTGAGTGTCCCTCTCCAGGCCTTGGCCTCTACTGGCCAGTGCTATGATATGTGCTAGCCTGCTACCTCCTATTAGCACATCCTGCTAGATGCTGTTCACTTTGCCCAGTTAGAAGCTCCTCTGTTGTAGGCAAGGTTAAGTAGGTTAGTGCTTGTGTTTGTATGTTCAGTTGCTCAGAGTGAAACTGTTTCTYTTTYTTTYCAGATCTGTAACATCATGAATGCTTCAGCTGATGAAATGTTCAACTTTCAGGTAATCTGCCTACCCATTTAATGAAACTGCATTTTACTAGAAAAATTTTGTTTCAAAAATGATGGATTGAATTAGGTTTTTCCACTTGAAACAACTTTTGGGGGGCTAGAGAGATAGGTCAGCAG
