# Supplementary material for: Revisiting the role of Dcc in visual system development with a novel eye clearing method
Source: eLife. 2020 Feb 25;9:e51275. doi: 10.7554/eLife.51275 (PMC7062470; doi:10.7554/eLife.51275)
Supplement: Supplementary file 4. [file elife-51275-supp4.docx]

| **Key Resources Table** | | | | |
| --- | --- | --- | --- | --- |
| **Reagent type (species) or resource** | **Designation** | **Source or reference** | **Identifiers** | **Additional information** |
| genetic reagent (include species here) | *Dkk3-Cre* | RIKEN BRC |  | (Sato et al., 2007) |
| genetic reagent (include species here) | *Dcc KO* | (Fazeli et al., 1997) |  |  |
| genetic reagent (include species here) | *Rosa^Tom^* | Jackson Laboratories |  |  |
| genetic reagent (include species here) | *Dcc ^Kanga^* | Jackson Laboratories | Stock no: 029220 | (Finger et al., 2002) |
| genetic reagent (include species here) | *Dcc flox* | Dr Anton Berns |  | (Krimpenfort et al., 2012) |
| genetic reagent (include species here) | *Netrin flox* | (Moreno-Bravo et al., 2018) |  |  |
| genetic reagent (include species here) | *Dkk3-Cre;Rosa^Tom^* | This paper | N/A | N/A |
| genetic reagent (include species here) | *Dkk3-Cre;Dcc lox* | This paper | N/A | maintained on C57BL/6J background |
| genetic reagent (include species here) | *Dkk3-Cre;Ntn1 lox* | This paper | N/A | maintained on C57BL/6J background |
| antibody | Rabbit polyclonal anti-Islet1 | Abcam | Cat# Ab20670; RRID: AB_881306 | 1:300 (IF) |
| antibody | Sheep polyclonal anti-Chx10 | Exalpha | Cat#; X1180-P  RRID: AB_2314191 | 1:300 (IF) |
| antibody | Mouse monoclonal anti-Calbindin | Swant | Cat# 300;  RRID: N/A | 1:1000 (IF) |
| antibody | Goat polyclonal anti-Chat | Abcam | Cat# Ab144b;  RRID: N/A | 1:500 (IF) |
| antibody | Rabbit polyclonal anti-Rbpms | Phosphosolution | Cat# 1830-RBPMS; RRID:AB_2492225 | 1:500 (IF) |
| antibody | Goat polyclonal anti-Dcc (A20) | Santa Cruz | Cat# sc-6535;  RRID:AB_2245770 | 1:500 (IF) |
| antibody | Rabbit polyclonal anti-Sox2 | Abcam | Cat# Ab97959; RRID:AB_2341193 | 1:500 (IF) |
| antibody | Mouse monoclonal anti-ßIII tubulin | Covance | Cat# MMS435P; RRID:AB_2313773 | 1:500 (IF) |
| antibody | Mouse monoclonal anti-Crx | Abnova | Cat# H1406-M02; RRID:AB_606098 | 1:2000 (IF) |
| antibody | Goat polyclonal anti-Pax2 | R&D systems | Cat# AF3364;  RRID:AB_10889828 | 1:300 (IF) |
| antibody | Rabbit polyclonal anti-Pax2 | Life technologies | Cat# 7160000; RRID:N/A | 1:300 (IF) |
| antibody | Goat polyclonal anti-Collagen IV | BioRad | Cat# 134001;  RRID:AB_2082646 | 1:400 (IF) |
| antibody | Goat polyclonal anti-Opn1sw | Santa Cruz | Cat# sc-14363; RRID:AB_2158332 | 1:1000 (IF) |
| antibody | Mouse monoclonal anti-Laminin | Abcam | Cat# ab11575; RRID:AB_298179 | 1:300 (IF) |
| antibody | Rabbit polyclonal anti-dsRed | Clontech | Cat# 632496;  RRID:AB_10013483 | 1:300 (IF) |
| antibody | Mouse monoclonal anti-Rhodopsin | Millipore | Cat# MABN15; RRID:AB_10807045 | 1:500 (IF) |
| antibody | Rabbit polyclonal anti-Recoverin | Millipore | Cat# AB5585;  RRID:AB_2253622 | 1:1000 (IF) |
| antibody | Rabbit polyclonal anti-Opn1mw | Millipore | Cat# AB5405;  RRID:AB_177456 | 1:300 (IF) |
| antibody | Rabbit polyclonal anti-Arrestin-C | Abcam | Cat# ab-15282; RRID:AB_1163387 | 1:1000 (IF) |
| antibody | Goat polyclonal anti-Contactin-2/TAG-1 | R&D Systems | Cat# AF4439; RRID:AB_2044647 | 1:500 (IF) |
| antibody | Donkey Anti-Rabbit IgG (H+L) Alexa Fluor 488 AffiniPure | Jackson ImmunoResearch Labs | Cat# 711-545-152; RRID:AB_2313584 | 1:500 (IF) |
| antibody | Donkey Anti-Rabbit IgG (H+L) Alexa Fluor 647 AffiniPure | Jackson ImmunoResearch Labs | Cat# 711-605-152; RRID:AB_2492288 | 1:500 (IF) |
| antibody | Donkey Anti-Rabbit IgG (H+L) Cy3 AffiniPure (min X Bov,Ck,Gt,GP,Sy Hms,Hrs,Hu,Ms,Rat,Shp Sr Prot) | Jackson ImmunoResearch Labs | Cat# 711-165-152; RRID:AB_2307443 | 1:500 (IF) |
| antibody | Bovine Anti-Goat IgG (H+L) Alexa Fluor 647 AffiniPure | Jackson ImmunoResearch Labs | Cat# 805-605-180 ;RRID:AB_2340885 | 1:500 (IF) |
| antibody | Donkey Anti-Goat IgG (H+L) Cy™3 AffiniPure | Life technologies | Cat# A21432 ; RRID:AB_141788 | 1:500 (IF) |
| antibody | Donkey Anti-Goat IgG (H+L) 488 | Life technologies | Cat# A11055 ; RRID:AB_2534102 | 1:500 (IF) |
| antibody | Donkey anti-Mouse IgG (H+L) Secondary Antibody, Alexa Fluor 488 | Life technologies | Cat# A-21202 RRID:AB_141607 | 1:500 (IF) |
| antibody | Donkey Anti-Mouse IgG (H+L) Alexa Fluor 647 AffiniPure | Jackson ImmunoResearch Labs | Cat# 715-605-150; RRID:AB_2340862 | 1:500 (IF) |
| antibody | Donkey Anti-Mouse IgG (H+L) Alexa Fluor 647 AffiniPure | Jackson ImmunoResearch Labs | Cat# 713-165-1471; RRID:N/A | 1:500 (IF) |
| antibody | Donkey Anti-Sheep IgG (H+L) Alexa Fluor 647 AffiniPure | Jackson ImmunoResearch Labs | Cat# 713-605-147; RRID:AB_2340751 | 1:500 (IF) |
| antibody | Donkey Anti-Sheep IgG (H+L) Alexa Fluor 488 AffiniPure | Life technologies | Cat# A11015; RRID:AB_141362 | 1:500 (IF) |
| sequence-based reagent | Forward-PDE6b^rd1^ | This paper | PCR primers | ctgcacacagacatccagtc |
| sequence-based reagent | Reverse-PDE6b^rd1^ | This paper | PCR primers | ccatgcctggctgaagttgt |
| chemical compound, drug | Dichloromethane (DCM) | Sigma-Adrich | Cat# 270997  CAS Number: 75-09-2 | N/A |
| chemical compound, drug | Dibenzyl ether (DBE) | Sigma-Adrich | Cat# 108014  CAS Number: 103-50-4 | N/A |
| chemical compound, drug | Dimethyl Sulfoxide | Sigma-Adrich | Cat# D8418-250mL  CAS Number: 67-68-5 |  |
| chemical compound, drug | Normal Donkey Serum | Millipore | Cat# S30-M | N/A |
| chemical compound, drug | Methanol (MeOH) | VWR Chemicals | Cat# 20847.360  CAS Number: 67-56-1 | N/A |
| chemical compound, drug | Gelatin | VWR Chemicals | Cat# 24350.262  CAS Number: 9000-70-8 | N/A |
| chemical compound, drug | Hydrogen peroxide solution (H_2_O_2_) | Sigma-Adrich | Cat# 216763  CAS Number: 7722-84-1 | N/A |
| chemical compound, drug | Thimerosal | Sigma-Adrich | Cat# T8784-5g  CAS Number: 54-64-8 | N/A |
| chemical compound, drug | Triton X100 | Sigma-Adrich | Cat# X100-500ml  CAS Number: 9002-93-1 | N/A |
| chemical compound, drug | TAE buffer | Invitrogen | Cat# 15558026 | N/A |
| chemical compound, drug | Agarose | Carl ROTH | Cat# 2267.4  CAS Number: 9012-36-6 | N/A |
| software, algorithm | Imspector software | LaVision Biotec |  | http://www.lavisionbiotec.com/ |
| software, algorithm | Imaris x64 software (version 9.1.2) | Bitplane | RRID:SCR_007370 | <http://www.bitplane.com/imaris/imaris> |
| software, algorithm | ImageJ (1.50e, Java 1.8.0_60, 64-bit) | NIH | RRID:SCR_003070 | <http://imagej.nih.gov/ij/>; |
| software, algorithm | iMovie (version 10.1.1) | Apple |  | http://www.apple.com/fr/imovie/ |
| software, algorithm | Graphpad Prism | Graphpad.com | Version 7.0 | Used for statistical analysis and graph generation |
| other | Hoechst | Sigma | Cat# B2883 |  |
| other | TO-PRO-3 | ThermoFischer | Cat# T3605 | N/A |
| other | Cholera Toxin Subunit B (Recombinant), Alexa Fluor™ 647 Conjugate | ThermoFischer | Cat# C34778 | N/A |
| other | Cholera Toxin Subunit B (Recombinant), Alexa Fluor™ 555 Conjugate | ThermoFischer | Cat# C22843 | N/A |

**References**

Fazeli A, Dickinson SL, Hermiston ML, Tighe R V, Steen RG, Small CG, Stoeckli ET, Keino-Masu K, Masu M, Rayburn H, Simons J, Bronson RT, Gordon JI, Tessier-Lavigne M, Weinberg RA. 1997. Phenotype of mice lacking functional Deleted in colorectal cancer (Dcc) gene. *Nature* **386**:796–804. doi:10.1038/386796a0,PMID:9126737

Finger JH, Bronson RT, Harris B, Johnson K, Przyborski SA, Ackerman SL. 2002. The Netrin 1 Receptors Unc5h3 and Dcc Are Necessary at Multiple Choice Points for the Guidance of Corticospinal Tract Axons. *J Neurosci* **22**:10346–10356. doi:10.1523/JNEUROSCI.22-23-10346.2002,PMID:12451134

Krimpenfort P, Song J-Y, Proost N, Zevenhoven J, Jonkers J, Berns A. 2012. Deleted in colorectal carcinoma suppresses metastasis in p53-deficient mammary tumours. *Nature* **482**:538–541. doi:10.1038/nature10790,PMID:22358843

Moreno-Bravo JA, Puiggros SR, Blockus H, Dominici C, Zelina P, Mehlen P, Chédotal A. 2018. Commissural neurons transgress the CNS/PNS boundary in absence of ventricular zone-derived netrin-1. *Development* **145**:dev.159400. doi:10.1242/dev.159400,PMID:29343638

Sato S, Inoue T, Terada K, Matsuo I, Aizawa S, Tano Y, Fujikado T, Furukawa T. 2007. Dkk3-Cre BAC Transgenic Mouse Line : A Tool for Highly Efficient Gene Deletion in Retinal Progenitor Cells. *Genesis* **45**:502–507. doi:10.1002/dvg,PMID:17661397
